# Advances in Extracellular Matrix-Associated Diagnostics and Therapeutics

**DOI:** 10.3390/jcm14061856

**Published:** 2025-03-10

**Authors:** Morten Karsdal, Thomas R. Cox, Amelia L. Parker, Nicholas Willumsen, Jannie Marie Bülow Sand, Gisli Jenkins, Henrik H. Hansen, Anouk Oldenburger, Kerstin E. Geillinger-Kaestle, Anna Thorsø Larsen, Darcey Black, Federica Genovese, Alexander Eckersley, Andrea Heinz, Alexander Nyström, Signe Holm Nielsen, Lucas Bennink, Lars Johannsson, Anne-Christine Bay-Jensen, Dana E. Orange, Scott Friedman, Mads Røpke, Vincent Fiore, Detlef Schuppan, Florian Rieder, Benjamin Simona, Lee Borthwick, Mark Skarsfeldt, Haakan Wennbo, Paresh Thakker, Ruedi Stoffel, Graham W. Clarke, Raghu Kalluri, Darren Ruane, Faiez Zannad, Joachim Høg Mortensen, Dovile Sinkeviciute, Fred Sundberg, Molly Coseno, Christian Thudium, Adam P. Croft, Dinesh Khanna, Michael Cooreman, Andre Broermann, Diana Julie Leeming, Ali Mobasheri, Sylvie Ricard-Blum

**Affiliations:** 1 Nordic Bioscience, 2730 Herlev, Denmark; nwi@nordicbio.com (N.W.); jsa@nordicbio.com (J.M.B.S.); atl@nordicbo.com (A.T.L.); fge@nordicbio.com (F.G.); shn@nordicbio.com (S.H.N.); acbj@nordicbio.com (A.-C.B.-J.); jhm@nordicbio.com (J.H.M.); dsi@nordicbio.com (D.S.); djl@nordicbio.com (D.J.L.); 2 Garvan Institute of Medical Research, Sydney 2010, Australia; t.cox@garvan.org.au (T.R.C.); am.parker@garvan.org.au (A.L.P.); 3 School of Clinical Medicine, St Vincent’s Clinical Campus, UNSW Medicine & Health, UNSW, Sydney 2010, Australia; 4 Margaret Turner Warwick Centre for Fibrosing Lung Disease, National Heart and Lung Institute, NIHR Imperial Biomedical Research Centre, Imperial College London, London SW7 2AZ, UK; gisli.jenkins@imperial.ac.uk; 5 Gubra, 2970 Hørsholm, Denmark; hbh@gubra.dk; 6 Global Drug Discovery, Novo Nordisk, 2760 Maaloev, Denmark; auog@novonordisk.com; 7 Department of Immunology and Respiratory Diseases Research, Boehringer Ingelheim Pharma GmbH & Co. KG, 88400 Biberach an der Riss, Germany; kerstin.geillinger-kaestle@boehringer-ingelheim.com; 8 TherapeutAix UG, 52062 Aachen, Germany; darcey@therapeutaix.com; 9 Wellcome Centre for Cell Matrix Research, Division of Musculoskeletal and Dermatological Sciences, School of Biological Sciences, University of Manchester, Manchester M13 9PL, UK; alexander.eckersley@manchester.ac.uk; 10 LEO Foundation Center for Cutaneous Drug Delivery, Department of Pharmacy, University of Copenhagen, 2100 Copenhagen, Denmark; andrea.heinz@sund.ku.dk; 11 Department of Dermatology, Faculty of Medicine, Medical Center—University of Freiburg, 79106 Breisgau, Germany; alexander.nystroem@uniklinik-freiburg.de; 12 3Helix, Inc., Salt Lake City, UT 84101, USA; lucas.bennink@3helix.com; 13 Antaros Medical AB, 752 37 Uppsala, Sweden; lars.johansson@antarosmedical.com; 14 Hospital for Special Surgery, The Rockefeller University, New York, NY 10065, USA; dorange@rockefeller.edu; 15 Icahn School of Medicine at Mount Sinai, 1425 Madison Avenue, New York, NY 10029, USA; scott.friedman@mssm.edu; 16 Novo Nordisk, 2860 Søborg, Denmark; omrp@novonordisk.com; 17 Boehringer Ingelheim, 55218 Ingelheim am Rhein, Germany; vincent.fiore@boehringer-ingelheim.com; 18 Institute of Translational Immunology, University Medical Center, Johannes Gutenberg University Mainz, 55131 Mainz, Germany; detlef.schuppan@unimedizin-mainz.de; 19 Department of Inflammation and Immunity, Cleveland Clinic Foundation, Cleveland, OH 44195, USA; riederf@ccf.org; 20 Ectica Technologies AG, 8005 Zürich, Switzerland; simona@ectica-technologies.com; 21 FibroFind Ltd., FibroFind Laboratories, Medical School, Newcastle upon Tyne NE2 4HH, UK; lee.borthwick@fibrofind.com; 22 Newcastle Fibrosis Research Group, Biosciences Institute, Newcastle University, Newcastle upon Tyne NE1 7RU, UK; 23 Takeda, Translational Medicine Biomarkers Gastrointestinal & Global, Boston, MA 02110, USA; hakan.wennbo@takeda.com (H.W.); paresh.thakker@takeda.com (P.T.); 24 Roche Diagnostics International Ltd., 6343 Rotkreuz, Switzerland; ruedi.stoffel@roche.com; 25 Translational Science and Experimental Medicine, Research and Early Development, Respiratory & Immunology, BioPharmaceuticals R&D, AstraZeneca, 431 83 Gothenburg, Sweden; graham.clarke1@astrazeneca.com; 26 School of Immunology and Microbial Sciences, Faculty of Life Sciences and Medicine, King’s College, London E1 9RT, UK; 27 Department of Cancer Biology, Metastasis Research Center, University of Texas MD Anderson Cancer Center, Houston, TX 77030, USA; kalluri@mdanderson.org; 28 Janssen Immunology, Translational Sciences and Medicine, La Jolla, CA 92037, USA; druane@its.jnj.com; 29 Division of Heart Failure and Hypertension, and of the Inserm CIC, University of Lorraine, 54000 Metz, France; f.zannad@chru-nancy.fr; 30 Sengenics Corporation LLC, Wilmington, DE 19801, USA; f.sundberg@sengenics.com (F.S.); m.coseno@sengenics.com (M.C.); 31 National Institute for Health and Care Research (NIHR) Birmingham Biomedical Research Centre, University of Birmingham, Birmingham B15 2TT, UK; a.p.croft@bham.ac.uk; 32 Institute of Inflammation and Ageing, Queen Elizabeth Hospital, University of Birmingham, Birmingham B15 2TT, UK; 33 Michigan Medicine, University of Michigan, Ann Arbor, MI 48109, USA; khannad@med.umich.edu; 34 Inventiva Inc., New York, NY 10001, USA; michael.cooreman@inventivapharma.com; 35 Department of CardioMetabolic Diseases Research, Boehringer Ingelheim Pharma GmbH & Co. KG, 88400 Biberach an der Riss, Germany; andre.broermann@boehringer-ingelheim.com; 36 Faculty of Medicine, University of Oulu, 90570 Oulu, Finland; ali.mobasheri@oulu.fi; 37 Department of Regenerative Medicine, State Research Institute Centre for Innovative Medicine, 08406 Vilnius, Lithuania; 38 Faculté de Médecine, Université de Liège, 4000 Liège, Belgium; 39 Department of Joint Surgery, First Affiliated Hospital of Sun Yat-sen University, Guangzhou 510080, China; 40 Institut de Chimie et Biochimie Moléculaires et Supramoléculaires (ICBMS), UMR 5246 CNRS, ICBMS, University Lyon 1, 69622 Villeurbanne Cedex, France; sylvie.ricard-blum@univ-lyon1.fr

**Keywords:** extracellular matrix, chronic diseases, drug development, liver disease, solid tumors, pharmacology congress

## Abstract

The extracellular matrix (ECM) is the common denominator of more than 50 chronic diseases. Some of these chronic pathologies lead to enhanced tissue formation and deposition, whereas others are associated with increased tissue degradation, and some exhibit a combination of both, leading to severe tissue alterations. To develop effective therapies for diseases affecting the lung, liver, kidney, skin, intestine, musculoskeletal system, heart, and solid tumors, we need to modulate the ECM’s composition to restore its organization and function. Across diverse organ diseases, there are common denominators and distinguishing factors in this fibroinflammatory axis, which may be used to foster new insights into drug development across disease indications. The 2nd Extracellular Matrix Pharmacology Congress took place in Copenhagen, Denmark, from 17 to 19 June 2024 and was hosted by the International Society of Extracellular Matrix Pharmacology. The event was attended by 450 participants from 35 countries, among whom were prominent scientists who brought together state-of-the-art research on organ diseases and asked important questions to facilitate drug development. We highlight key aspects of the ECM in the liver, kidney, skin, intestine, musculoskeletal system, lungs, and solid tumors to advance our understanding of the ECM and its central targets in drug development. We also highlight key advances in the tools and technology that enable this drug development, thereby supporting the ECM.

## 1. Introduction

**Figure 1:**
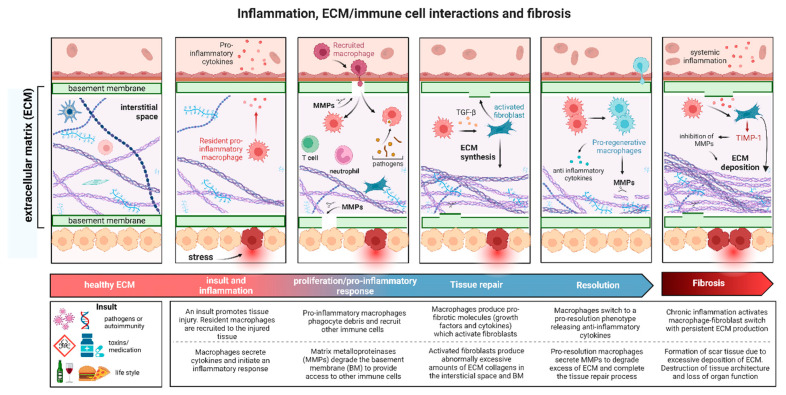
Interactions between the ECM and immune cells are the key drivers behind inflammatory response, regular tissue repair, and fibrosis development mechanisms. Modified from Zawadzki et al. [5].

The ECM is a complex and dynamic network of proteins and polysaccharides that provides structural and biochemical support to surrounding cells. It plays a critical role in tissue architecture, cellular function, and signaling. The ECM regulates essential cellular processes such as cell adhesion, migration, differentiation, and survival, and is involved in tissue repair, organ development, and homeostasis. The ECM is controlled by a balance between the rates of formation and degradation of extracellular macromolecules, predominantly collagens, which provide structural support to multicellular tissues and are essential to several biological pathways, particularly in tissue repair [1,2,3,4]. Interactions between the ECM and cells are crucial in promoting successful tissue repair and sustaining a chronic inflammatory environment that can produce irreversible changes in the ECM, which then lead to the development of pathologies (see Figure 1).

Tissue injury due to bacterial and viral infections, toxins, metabolic dysregulation, autoimmune reactions, or mechanical stress disrupts ECM homeostasis across its various compartments, initiating damage to the basement membrane that propagates to the interstitial space [2]. Damage to endothelial or epithelial cells initially induces the release of proteolytic enzymes that degrade the extracellular matrix and basement membrane, providing immune cells access to the injured tissues. The basement membrane is a scaffold composed of a network of collagen IV, laminins, nidogens, and proteoglycans that provide structural support to endothelial and epithelial cells and protection against mechanical stress. A degraded basement membrane recruits immune cells such as macrophages to the injured site, initiating an inflammatory response by secreting proinflammatory molecules, including cytokines and growth factors [3]. These proinflammatory molecules send profibrotic signals to (myo-)fibroblasts located in the interstitial ECM, where they synthesize large amounts of collagen, predominantly collagen types I and III, to restore the structure and functional integrity of the damaged tissue. The interplay between macrophages and (myo-)fibroblasts plays a crucial role in orchestrating tissue repair; as such, abnormal biological processes that allow inflammation to further progress can lead to fibrosis. Under normal conditions, the final step of the tissue repair process is characterized by the anti-inflammatory phase, in which pro-resolution macrophages produce proteolytic enzymes to remove excess collagenous fibrotic tissue produced by activated [6]. However, during persistent inflammatory processes and pathological conditions, the tissue repair process is not completed, and ECM homeostasis is chronically disrupted by excessive formation or degradation of collagen, resulting in “wounds that do not heal” and progressive damage to the tissue architecture, ultimately leading to loss of organ function. Therefore, investigating ECM composition and ECM–cell interactions is critical to understanding the mechanisms behind chronic diseases such as fibrosis and to guiding future treatment.

ECM2024 took place in Copenhagen, Denmark, in June 2024, and was hosted by the International Society of Extracellular Matrix Pharmacology. The event brought together 450 participants from 35 countries and included 175 abstracts, 85 speakers, and 30 sponsors. Following the successful debut of ECM2022, ECM2024 continued to emphasize the crucial role of the ECM in more than 50 chronic diseases that affect the musculoskeletal system and organs such as the liver, heart, kidneys, skin, gut, and lungs. There is a continued need for ECM-focused pharmacology to develop effective treatments tr halt or reverse tissue damage and organ function decline. Oral and poster sessions facilitated in-depth discussions and networking, complemented by industry-sponsored symposia showcasing the latest advancements in ECM-targeted therapies. The congress focused on target discovery and drug development, aiming to integrate knowledge across medical specialties and accelerate new treatments. ECM2024 underscored the power of interdisciplinary collaboration in ECM research, aiming to improve patient outcomes in chronic diseases. It also highlighted the ongoing need for exploration and innovation in ECM pharmacology to address the complexities of managing these disease conditions.

- Common mechanisms of fibrosis: Studies explored the common molecular pathways underlying fibrosis across various organs and diseases. An emphasis was placed on mechanisms of tissue formation by activated fibroblasts and immune cell–mediated tissue destruction as a central component of the fibroinflammatory axis.
- Fibroblast action and heterogeneity: The importance of fibroblast activation, ECM production and deposition for clinical outcomes, and the heterogeneity of fibroblast function were discussed.
- Genetic and molecular markers: Research highlighted findings on genetic and molecular markers implicated in fibrosis and related chronic diseases.
- Therapeutic approaches: Developments included novel treatments targeting fibrosis, drug testing, potential biomarkers for early diagnoses, monitoring of disease progression, pharmacodynamic effects, and intervention efficacy.
- Disease models: Advancements in the development and utilization of animal models to study fibrosis and ECM remodeling across organs were showcased. In vitro and ex vivo technologies that support and advance drug development were highlighted.

These proceedings present 21 areas of high interest, beginning with a focus on individual organs, in vitro and in vivo models, and proteomics, followed by an exploration of key topics that enhance our understanding of cellular components and ECM interactions in organ pathology development.

## 2. The ECM in Lung Diseases: Genetics, Mechanisms, and Biomarkers of Interstitial Lung Diseases

Interstitial lung diseases (ILDs) encompass a broad spectrum of lung conditions that share common features of lung scarring and breathlessness [7]. ILDs can be categorized into several subgroups, with the main categories being idiopathic, autoimmune-related, and exposure-related ILDs. Autoimmune-related ILDs are often associated with connective tissue diseases such as systemic sclerosis (SSc) and rheumatoid arthritis (RA). Exposure-related ILDs may result from inhaling organic particles or undergoing chemotherapy. Idiopathic pulmonary fibrosis (IPF) is the most common and aggressive form of ILD, characterized by irreversible lung scarring and a median survival of only three to five years post diagnosis. Currently, two therapies—nintedanib and pirfenidone—are available to slow the decline in lung function, but these treatments are limited by significant adverse effects and do not effectively halt or reverse disease progression. Therefore, there is a significant unmet need for earlier diagnosis and more tolerable therapies that can halt disease progression or ultimately restore the function of fibrotic lung parenchyma.

In this context, leaders in pharmacology met to discuss novel insights into genetic drivers, disease pathology, novel treatment strategies, and the identification of potential diagnostic and/or prognostic biomarkers.

Genetic variants linked to disease progression and biological pathways provide a unique opportunity for the development of novel treatment strategies. In 2011, a single-nucleotide polymorphism in the mucin 5B (MUC5B) promoter was identified, demonstrating a substantially increased risk of developing IPF, which was independently replicated by numerous investigators [8]. The variant alone, however, is not sufficient to cause disease and is not necessary for disease development, indicating that additional genetic or environmental factors contribute [8]. Recently, whole-genome sequencing was performed on 958 patients with PF from two independent cohorts, identifying 90 genes that harbor significantly rarer variants compared with controls. Variants were classified as loss-of-function, missense, protein-altering, or protein-truncating. Interestingly, one of the top hits in both cohorts was hemicentin-1 (HMCN1), a member of the fibulin family [9]. HMCN1 is an ECM component that plays a critical role in the actin cytoskeleton, cellular adhesion, and the TGF-β signaling pathway [9,10]. TGF-β, Transforming Growth Factor Beta, is a family of signaling molecules (cytokines) that play an essential role in regulating various cellular processes, such as cell growth, differentiation, immune function, and tissue repair. In addition, several other rare variants were identified and linked to altered gene expression in the context of disease, including ECM genes COL6A1 and ADAMTSL1, which encode the alpha1 chain of type VI collagen and ADAMTS-like protein 1, respectively, providing a deeper understanding of genetic drivers and disease pathology [9]. Although significant advances have been made in identifying genetic variants that might contribute to disease development, a definitive link to clinical outcomes has not yet been established. Therefore, the regular inclusion of genetic testing in future clinical trials could increase our understanding of disease causality and aid in identifying novel endotypes.

In the past decade, technical advances in sequencing techniques, including spatial transcriptomics, have greatly improved our understanding of ILD/IPF pathology. Although fibroblast activation and its associated ECM deposition were historically central to IPF research, additional cell types, such as epithelial and endothelial cells, have recently emerged as potential therapeutic targets. To date, recurring epithelial injury has been considered the root cause of aberrant repair mechanisms, leading to self-perpetuating fibrosis, ECM deposition, lung stiffening, alveolar collapse, and eventual loss of functional lung parenchyma. Novel findings suggest that in cases of insufficient basement membrane repair, subsequent epithelial injury is associated with worse outcomes in IPF. Although alveolar epithelial cells attempt to regenerate injured alveolar structures, precursor cells appear to be halted in an intermediate or transitional state, with altered collagen expression profiles identified through single-cell sequencing [11], further contributing to basement membrane regeneration failure.

In addition to novel sequencing techniques, such as single-nucleus sequencing, proteome analysis can provide valuable insights into molecular mechanisms and reveal potential biomarkers linked to disease progression and specific patient populations to support precision medicine. Overlapping proteomic profiles between independent cohorts strengthen the applicability of biomarkers for interventional trials. This was highlighted in a presentation of novel proteomics data from Novartis, which, in line with recent studies, identified specific autoantibodies and proteins, such as surfactant protein B, associated with disease progression [12,13].

Collecting biomarker and proteomics data in larger cohorts can not only facilitate the identification of disease progression biomarkers but also allow for patient stratification in precision medicine.

TGF-β signaling is a major contributor to the development of fibrosis across organs and, therefore, has been targeted by multiple approaches, such as interference with integrins, mTORC1, galectin-3, or the inhaled TGF-β type I receptor (ALK5) inhibitor (ALK5i) [14]. Some compounds targeting TGF-β signaling have already reached clinical evaluation, while new targets interfering with TGF-β-driven biology are emerging. Galectin-3 was found to induce TGF-β1 activation by colocalizing with integrins—particularly the αvβ1 integrin—and the TGF-β receptor. This facilitated clustering of the integrin with the TGF-β receptor, which enabled the spatial colocalization required for TGF-β activation. This colocalization was only evident in the lung fibroblasts of IPF patients, indicating that the proteins exhibited a stronger inherent association in the diseased state [15]. Bexotegrast, a dual integrin (αvβ6 and αvβ1) inhibitor developed by Pliant, and data from a Phase 2a clinical trial demonstrate an impact on TGF-β signaling in fibrogenic cell populations, resulting in improved forced vital capacity, a reduction in collagen I in the lungs according to positron emission tomography (PET) imaging, and changes in PRO-C3 levels in the plasma.

Changes in ECM organization and composition also play a large role in conditions like emphysema. Therefore, expanding our knowledge of cell type-specific ECM-related gene expression, the three-dimensional organization of the matrix, and its interactions with different cell types are key to treating lung diseases such as ILD.

## 3. Cardiovascular and Renal ECM Research

Biomarkers reflecting the formation (PINP, procollagen carboxyterminal propeptide (PICP), the N- and C-terminal propeptides of procollagen I) and degradation (CITP) of collagen type I (COL1) have been used for decades in cardiovascular diseases (CVDs). PICP and, to a limited extent, CITP have consistently shown promising results in identifying patients with increased cardiac fibrosis and with a higher likelihood of both progressing in the disease and responding to treatment [16,17,18]. However, none of these biomarkers are currently implemented in clinical biochemistry, and no systematic evaluation of their prognostic or pharmacodynamic ability has been undertaken. Moreover, the literature presents contrasting evidence on the usefulness of these biomarkers across different CVDs because they seem to be mostly relevant in heart failure cohorts. The lack of consistent results may be due to the use of different assays in detecting these analytes. It is apparent that COL1, even though it is the most abundant collagen in the human body, fails to encompass the complex involvement of ECM proteins in CVDs. Moreover, another isoform of COL1 may be more disease-relevant, and biomarkers quantifying a specific isoform may be more consistently related to disease progression and responses to treatment. For example, the homotrimer of COL1, which contains three α1 chains instead of two α1 and one α2 chains and which is of relevance in pancreatic cancer, also needs to be evaluated in other disease settings, such as tissue fibrosis [19].

It has been reported that modifications of the ECM in CVDs go beyond collagen I. Several collagens have altered turnover dynamics in different cardiovascular manifestations [19,20,21]. One of the collagens that has accumulated the highest amount of evidence in cardiovascular and renal diseases in the past decade is collagen type VI (COL6). The biomarker of COL6 formation, PRO-C6, can also quantify the profibrotic and proinflammatory COL6A3-derived molecule endotrophin, which has been shown to be a common biomarker of the risk of chronic kidney disease (CKD) and CVD. PRO-C6 was recently associated with an increased risk of mortality in several chronic diseases in a meta-analysis including more than 15,000 patients [22,23] This suggests that this biomarker may reflect a common mechanism involved in the progression of chronic fibroproliferative diseases, potentially due to its ability to detect endotrophin. Endotrophin may play an active role in triggering the processes that lead to organ failure and death, regardless of the initial etiology [23]. Novel data on PRO-C6 as a biomarker of long-term risk of major cardiovascular events in individuals with type 2 diabetes (T2D) were presented at the conference, confirming a robust, independent association between levels of circulating PRO-C6 and outcomes. Endotrophin may be a strong biomarker of risk because of its involvement in dysregulated wound healing processes. A close interaction between COL6 and platelets is suggested by both the platelet-derived growth factor, the main cytokine promoting COL6 production by fibroblasts, and the structure of COL6 domains, which resemble the attachment sites of the von Willebrand factor, the main platelet recruiter [24]. Canstatin, a known bioactive molecule derived from the cleavage of the α2 chain of COL6, has been examined as a biomarker of cardiovascular events and all-cause mortality in patients with advanced atherosclerosis, showing an independent association with the risk of outcome [25]. This novel evidence expands the current knowledge of canstatin beyond its anti-angiogenic potential.

Novel data were presented in [26] on the measurement of COL1 turnover, specifically on a fragment of COL1—C1M—as a biomarker of post-myocardial infarction mortality risk. This adds to the evidence of the potential of this biomarker as a predictor of outcome, as previously reported in patients with atherosclerosis [27] and in older adults [28,29].

A novel fragment of isomerized type III collagen (C3M iso) has also been introduced as a biomarker of CKD progression in T2D, as presented by Chrysoulidou and colleagues at ECM2024. Levels of this fragment, containing an isomerized amino acid and reflecting the turnover of long-lived type III collagen, are associated with CKD progression. This biomarker has the potential to add granularity to the ongoing tissue remodeling process because it detects post-translational isomerization that accumulates over time in tissues, specifically identifying the degradation of older collagen.

A note of caution regarding the indiscriminate use of collagen biomarkers in males and females is provided by urinary peptidomic data in CKD patients, as presented by Mina and colleagues at ECM2024, showing sex-specific differences in the collagen peptides identified in urine. For instance, a gender-associated difference in the abundance of COL1A2 peptides suggests sex-specific differences in ECM remodeling.

### Therapeutic Approaches

Regarding atherosclerosis, data on the contribution of hypoxia to changes in the arterial ECM were presented. Coronary artery endothelial cells exposed to hypoxia increased versican production, which reduced cell adhesion, enhanced proliferation, and increased the ability to bind myeloperoxidase. Myeloperoxidase generates hypochlorous acid, inducing arterial wall damage, as shown in an oral presentation at the conference. The authors showed that heparin treatment disrupted myeloperoxidase binding, suggesting that this could be a therapeutic approach.

In aortic stenosis, a fibrotic and calcified aortic valve often causes increased mortality in patients. Data show that TGF-β1 induces KCa3.1 (intermediate conductance calcium-activated potassium channel protein 4, KCNN4) channel expression in aortic valve tissue fibroblasts in patients with aortic stenosis. Treatment with KCa3.1 channel blocker senicapoc reduces ACTA2 and COL1A1 mRNA expression, suggesting that blocking KCa3.1 may offer an unexplored therapeutic avenue for aortic stenosis.

Ferroptosis has been identified as an enriched pathway in the proteomic analysis of cryoinjured living myocardial rat slices, a model that reflects heart failure characteristics, as demonstrated by Fiedler and colleagues at ECM2024. Inhibiting ferroptosis with ferrostatin in an ex vivo model resulted in the attenuation of profibrotic processes, promising a new therapeutic target for heart failure.

Aloxistatin, an inhibitor of cathepsin G, has been tested for its antifibrotic activity in human cardiac fibroblasts and rat myocardial slices, as demonstrated by Jordan and colleagues at ECM2024. Analyses of fibroblasts showed reduced migratory activity and expression of profibrotic markers. In myocardial slices, miR-21 expression was reduced, and cardiotoxicity was absent; further gene expression analyses will be performed to confirm the potential of repositioning this drug for heart fibrosis treatment.

## 4. Advances in ECM Pharmacology in Inflammatory Bowel Disease

Advances in our understanding of the interplay between inflammation and fibrosis, which induces fibro inflammation, have opened new avenues for therapeutic intervention based on novel mechanisms of action. This includes existing drugs, which were primarily thought to target inflammation but may also possess antifibrogenic properties [30]. This is important because although treating inflammation in inflammatory bowel disease (IBD) is crucial, it may not be sufficient to prevent fibrosis progression. This is demonstrated by the poor response to biologics, which has been linked to intestinal fibrosis [31]. Despite the increased use of biologics and immunosuppressants, the rate of surgical intervention has not markedly declined [32]. Therefore, when considering ECM-directed therapies, it is essential to account for factors such as inflammation, tissue damage, and fibrosis [30]. These factors contribute to long-term complications, such as strictures with intestinal obstruction and fistulas, which can lead to the need for surgery and reduced quality of life [32]. The ceiling effect of achieving mucosal healing or durable remission [33] suggests that by addressing mechanisms of ECM remodeling or fibrosis, we may enhance our chances of preventing disease progression and achieving comprehensive, lasting remission in patients [30,34]. Type III and VI collagens are highly associated with intestinal fibrosis, with the alpha-3 chain of type VI collagen (COL6A3) being particularly elevated in intestinal tissues from ulcerative colitis (UC) and Crohn’s disease (CD) patients [35]. Quantification of the alpha-3 chain of collagen VI in serum via PRO-C6 reveals elevated levels in pediatric CD patients with magnetic resonance elastography (MRE)-confirmed stenosis, consistent with previous findings that PRO-C6 is a marker of severe fibrogenesis [36]. Recent scRNAseq studies in IBD have highlighted the role of various stromal cellular subsets within the disease mucosa, including inflammatory activated fibroblasts (IAFs), ADAM-like protein decysin-1 (ADAMDEC1+) fibroblasts, and myofibroblasts [31]. These stromal subsets act as immunological hubs in IBD, indicating inadequate responses to anti-inflammatory therapies, and are implicated in the progression to complicated disease and stricture [37]. IAFs are functionally distinct and possess transcriptional machinery associated with ECM remodeling and fibroimmune pathways, representing a novel therapeutic target. Furthermore, the phenomenon known as creeping fat may play a significant role in the progression of CD strictures. This is likely relevant because creeping fat is associated with smooth muscle hyperplasia, the strongest contributor to luminal narrowing in CD. The interaction between creeping fat and the inflamed muscularis propria may exacerbate this process. Adipose tissue releases proinflammatory cytokines, profibrotic mediators, and factors that stimulate smooth muscle hyperplasia, contributing to wall thickening in stricturing CD [30,38]. As such, creeping fat in CD may not just be a byproduct of inflammation but also an active participant in the development of intestinal strictures, worsening the course of IBD and complicating its management [39]. Thus, the coming years will be crucial, as ECM pharmacology is a rapidly advancing field in IBD research.

### 4.1. Recent Highlights in ECM-Targeted Therapeutics

#### 4.1.1. Integrin Antagonists

Integrins are transmembrane receptors that mediate cell–ECM interactions and play a pivotal role in leukocyte trafficking to inflamed gut tissue. Vedolizumab, an α4β7 integrin antagonist, has already demonstrated efficacy. Although the primary function of integrin antagonists is to reduce inflammation, their ability to limit immune cell infiltration may also slow the progression of fibrosis by mitigating the continuous cycle of ECM damage and repair. This was previously demonstrated when vedolizumab reduced surrogate markers of mucosal damage and tissue degradation/formation ratios, as measured by MMP-mediated collagen III degradation (C3M) and collagen III formation (PRO-C3), collectively referred to as C3M/PRO-C3 [37]. Interestingly, the vedolizumab response was associated with a significant reduction in tissue IAFs, as confirmed by spatial transcriptomics approaches [40]. Furthermore, IBD patients with poor responses to vedolizumab maintained a robust IAF transcriptional profile within the diseased mucosa [29,31,41,42,43], reflecting continued tissue remodeling and immune cell recruitment. Next-generation integrin inhibitors focusing on integrin–ECM interactions may offer more effective control over both inflammation and fibrotic processes. For example, αvβ6 antagonists have been shown to be involved in the regulation of TGF-β activity.

#### 4.1.2. JAK Inhibitors

Janus kinases (JAKs) are a family of intracellular tyrosine kinases that play a crucial role in cytokine signaling. The impact of JAK inhibitors on the ECM in IBD is an emerging area of interest. Several mechanisms of JAK inhibitors may influence ECM dynamics. Multiple interleukins signal through the JAK-STAT pathway and are key drivers of ECM remodeling in IBD. By inhibiting JAKs not only in immune cells but also in fibroblasts, these signaling pathways may be blocked [44] thereby decreasing matrix metalloproteinase (MMP) activity and reducing ECM degradation and mucosal damage [45]. This was confirmed in the Phase 2 Fitzroy study, where filgotinib, a JAK inhibitor, demonstrated a reduction in mucosal damage quantified by C4M and C3M in CD patients who responded to the drug [45]. The JAK-STAT pathway is involved in fibrogenesis, with cytokines such as IL-13 [45,46] and TGF-β promoting the differentiation of fibroblasts into myofibroblasts, which then produce excessive ECM components [47,48,49,50]. JAK inhibitors may help modulate this fibrotic response by downregulating the signaling pathways that drive fibroblast activation and ECM production. Although the canonical TGF-β pathway signals through ALK5 and SMADs, recent data have demonstrated that tofacitinib can reduce type III collagen production in vitro in fibroblasts, as quantified by PRO-C3 [51].

#### 4.1.3. Therapeutic Targeting of ECM Remodeling Pathways

Therapies targeting fibrogenic pathways, such as Rho-associated coiled-coil-containing protein kinases (ROCKs) [52] and ALK5 inhibitors, as well as therapeutics like TL1A, which modulate the fibroinflammatory axis, could potentially prevent or reverse fibrosis. Advances in this area have been bolstered by new insights from high-resolution imaging and omics technologies, which have provided a more detailed understanding of ECM dynamics in IBD [30,51].

ROCK proteins regulate various cellular functions, including cell shape, motility, and contraction, primarily through their effects on the actin cytoskeleton [53]. ROCK inhibitors can reduce fibroblast activation and myofibroblast differentiation, thereby limiting ECM deposition and the progression of fibrosis. ROCK signaling contributes to epithelial barrier dysfunction by promoting stress fiber formation and tight junction disassembly. Thus, ROCK inhibitors may also help restore epithelial barrier function, thereby reducing intestinal permeability and subsequent inflammation [52].

Recent clinical data from the 12-week induction Phase 2 ARTEMIS-UC and APOLLO-CD studies, which evaluated anti-TL1A in biologically naïve and biologically experienced populations, have demonstrated a significant reduction in tissue signatures associated with remodeling. Moreover, reductions in the expression of individual genes, including *MMP3*, *MMP9*, and *COL1A1,* have also been reported, along with a decrease in the proportion of IAFs within diseased tissue. Further clinical evidence, including long-term follow-up, is needed, but these preliminary data reflect localized tissue remodeling changes associated with anti-TL1A therapy [54]. Additional clinical and biomarker data are necessary to assess its impact on ECM deposition, tissue remodeling, and progression to strictures.

#### 4.1.4. Neutrophil Extracellular Traps (Nets) and Involvement in Fibrosis

In IBD, excessive or dysregulated NET formation contributes to chronic inflammation and tissue damage by activated neutrophils [54,55]. A Specific Calprotectin Neo-epitope (CPa9-HNE) biomarker, which quantifies a calprotectin fragment generated from human neutrophil elastase-mediated degradation, is a useful tool for monitoring neutrophil activation and NETosis, as both calprotectin and elastase are NET components [56,57]. In IBD, NETs exacerbate mucosal injury by promoting the release of proinflammatory cytokines and activating other immune cells [58,59]. They also promote thrombosis and fibrosis, further complicating disease progression [60]. As a result, NETs are emerging as a potential therapeutic target in IBD, with research exploring strategies to regulate NET formation and reduce their harmful effects.

Recent advances in ECM pharmacology have highlighted the ECM as more than just a passive bystander in IBD, partly due to the high rate of severe intestinal fibrosis in IBD patients who respond poorly to anti-inflammatory therapies. Targeting ECM components and their interactions with immune cells presents a novel therapeutic strategy to complement existing treatments. However, the complexity of ECM biology demands a nuanced approach to advancing IBD treatments, requiring further research to translate these findings into safe and effective therapies.

The future integration of ECM-targeted therapies with precision medicine approaches, including the monitoring of ECM remodeling, holds great promise. Additionally, evaluating monotherapies that target the stromal–immune axis, alongside combination therapy approaches, may further influence ECM remodeling and stricture formation. Tailoring antifibroinflammatory treatments to individual patients could lead to better clinical outcomes, ultimately improving quality of life and reducing the need for surgery due to disease complications.

## 5. Quantifying Fibrolytic and Fibrogenic Activity in Rheumatic Diseases

The balance between fibrolysis and fibrogenesis in rheumatic diseases is not well understood and may play a key role in disease pathogenesis, as well as in how anti-inflammatory drugs alleviate symptoms and slow progression.

Although tissue degradation (fibrolysis) is well characterized [61,62] the abnormal accumulation of the ECM (i.e., fibrosis) has emerged as a pivotal yet often underappreciated factor in rheumatic diseases [63]. Previously considered a late-stage consequence of chronic inflammation, fibrosis is now recognized as a distinct pathological process that can significantly influence disease progression and patient outcomes.

In rheumatology, fibrosis manifests as joint stiffness, organ scarring, and functional decline. This dual burden of inflammation and fibrosis presents a significant therapeutic challenge. Although traditional treatments primarily target inflammation, the growing recognition of fibrosis as a distinct pathological process necessitates a more comprehensive approach. One of the major challenges in managing rheumatic diseases is treatment resistance, which can limit the effectiveness of available therapies and lead to poor outcomes. This resistance may be linked to fibrotic signatures that are not affected by current anti-inflammatory treatment regimens [60]. Deciphering the intricate interplay between inflammation and fibrosis is essential for the development of targeted therapies that can effectively halt disease progression and improve patients’ quality of life.

The multifaceted nature of fibrogenesis and fibrolysis, along with their complex interactions with inflammatory pathways, highlights the need for innovative strategies to address this critical aspect of rheumatic diseases.

Soluble biomarkers may offer a powerful tool for examining the intricate nature of diseases, particularly the balance between tissue breakdown (fibrolysis) driven by inflammation and tissue scarring driven by fibrogenesis. Fibro-associated soluble biomarkers could quantify disease activity at the tissue level by assessing the concentration of collagen fragments released from the tissue into circulation [64].

Tissue-associated markers that measure proteolytic fragments of collagens have been studied for over two decades. One of the first markers, CTX-I, is a cathepsin K-generated fragment of collagen I that is released during bone resorption. Data from one study [64] showed that CTX-I can predict bone mass changes in osteoporosis patients treated with alendronate as early as three months, while changes were not detectable on X-ray until 12 months [65]. Since then, the field has evolved, with additional markers now available to measure tissue fibrolysis.

Collagen degradation is particularly relevant in the context of rheumatic diseases because collagen is the major extracellular protein of bone and the interstitial matrix. Both AST and ALT, which are substantially affected by inflammation, are important markers in this process. Another marker of type I collagen is C1M [61,63], which, unlike CTX-I, is released from the interstitial matrix through MMP activity. Interestingly, C1M is significantly elevated compared to reference levels across various autoimmune diseases, including RA, Sjögren’s disease, lupus, and scleroderma, and it is correlated with disease activity scores indicative of widespread tissue inflammation across organs and pathologies [66]. Moreover, C1M may serve as a pharmacodynamic marker, responding to therapies such as anti-IL6, anti-IL1, and anti-tumor necrosis factor (TNF) inhibitors, as well as JAK inhibitors in RA, PSA, and AS, and to anti-IL17 and anti-IL23 in PSA [67,68,69,70]. Similar pharmacodynamic effects have been observed for other markers, including fragments of type III and IV collagens (C3M and C4M), which are released from the interstitial matrix and basement membranes, respectively [71].

Fibrogenesis remains a less understood component of rheumatic diseases. It can be assessed by the PRO-C3 biomarker, which reflects fibroblast activity and ECM formation. A recent study found that ECM markers such as C3M and C4M were reduced in patients with scleroderma and systemic sclerosis (SSC) following treatment with anti-IL6 drug tocilizumab [67,69]. However, PRO-C3, which is elevated in SSC above reference levels, remained unchanged throughout the course of treatment. This finding suggests, in line with the trial results, that tocilizumab has a limited effect on fibrosis. This is further supported by Madsen et al., who observed that high levels of PRO-C3 were associated with lower response rates [62].

In conclusion, fibrogenesis and fibrolysis are pathological processes involving the excessive remodeling of the ECM in various tissues, which can exacerbate the progression and outcomes of rheumatic diseases. Soluble biomarkers, such as those reflecting collagen degradation and formation, offer a powerful tool for investigating the complex nature of these diseases and the balance between tissue breakdown (fibrolysis) and tissue scarring (fibrogenesis). These biomarkers may not only quantify disease activity at the tissue level but also serve as indicators of disease activity, therapeutic response, and prognosis.

### 5.1. A Role for Synovial Fibroblasts in Mediating Chronic Pain Sensitization

Up to 20% of patients with RA are considered “difficult-to-treat”, meaning that they do not respond to multiple immunosuppressive agents with varying mechanisms of action [72,73,74]. Recent advances in transcriptomic profiling have revealed considerable variability in RA synovitis across patients. This has led to several clinical trials exploring whether synovial pathotypes could help select the most suitable immunosuppressive agent for each patient. A consistent finding from these efforts is that patients with synovium characterized by a paucity of inflammatory infiltrates and robust expression of a canonical fibroblast transcriptional signature—referred to as the “low inflammatory” [75], “pauci-immune” [76], “fibroblast cell-type abundance phenotype (CTAP)” [77] or “fibroid” [78]—are less likely to respond to disease-modifying antirheumatic drugs or biological therapies. This is concerning because up to half of synovial tissue samples from patients with longstanding RA undergoing arthroplasty [75] and one-third of synovial biopsy samples from early, untreated RA [79] exhibit fibroid synovium. This may help explain why up to 40% of RA patients report persistent pain [80]. A recent analysis of RA patients with fibroid synovium identified a module of 815 genes potentially associated with patient-reported pain in both established and early untreated RA [81,82].

A comparison of pain-associated gene expression from the adenosine monophosphate (AMP) phase 1 sorted bulk synovial B cells, fibroblasts, monocytes, T cells, and single-cell RNA sequencing data revealed that lining CD55+ fibroblasts exhibit the highest levels of pain-associated genes. The top pathways enriched in this pain-associated module included nervous system development and neurogenesis, featuring neurotrophin and axon guidance family members such as semaphorins, netrins, and ephrins. Additionally, fibroblast supernatants from fibroid RA synovium enhanced the growth of nociceptors in vitro [81]. These findings align with recent studies on fibroblasts from degenerate intervertebral discs [83,84,85] and painful osteoarthritic synovium [86] which also secrete factors that promote neuron growth in vitro and in vivo. These fibroblasts express gene signatures enriched in neuron outgrowth pathways. Specifically, degenerated disc fibroblasts have been shown to augment the growth of human-induced nociceptors in vitro and induce pain behaviors and neuron sprouting when injected intradiscally in rats. Collectively, these studies suggest that joint-resident fibroblasts likely play a crucial role in peripheral sensitization, highlighting the need for further investigation of these cells as potential treatment targets.

### 5.2. Role of Synovial Fibroblasts in Inflammation, Damage, and Repair

Fibroblasts have traditionally been regarded as homogeneous structural support cells responsible for the synthesis and modification of the ECM [87]. However, recent single-cell transcriptomic and spatial profiling studies have uncovered significant heterogeneity within fibroblasts. These cells exist in specialized states and are strategically positioned in distinct microanatomical niches within tissues [88]. As a result, fibroblasts are uniquely positioned to orchestrate the inflammatory response, controlling both its intensity and duration [89].

In RA, a prototypic immune-mediated inflammatory disease characterized by chronic inflammation and progressive joint damage, the synovium serves as the primary target tissue during the effector phase of the disease [90]. In its healthy state, the synovium consists of a protective barrier formed by tissue-resident macrophages in the lining layer, supported by a thin layer of fibroblasts. These components are situated on adipose-rich, loose connective tissue [87,88]. However, chronic joint inflammation is associated with significant architectural remodeling of the synovium, leading to the formation of highly organized sublining tissue. In this altered state, adipocytes are depleted, fibroblasts are expanded and activated, and dense aggregations of infiltrating immune cells emerge [89,90].

Fibroblasts act as the key conductors of the “immunological orchestra”, playing a central role in either suppressing or amplifying inflammatory processes, thereby determining the outcome of joint inflammation [82]. The transcriptional states of fibroblasts in the inflamed synovium have been defined to identify pathogenic fibroblasts with non-overlapping effector functions, which regulate inflammation or tissue damage [82]. During inflammation, lining-layer synovial fibroblasts lose their barrier phenotype, adopting a destructive program regulated by transcription factor ETS1. This program is characterized by the production of matrix metalloproteinases (MMPs) that degrade articular cartilage and bone while also inducing osteoclastogenesis through receptor activator of nuclear factor kappa-B ligand (RANKL) [91,92]. In contrast, sublining-layer fibroblasts differentiate in response to endothelially derived Notch signaling, acquiring immune effector functions that promote the recruitment and retention of inflammatory cells in the joint [82,91]. More recently, we and other researchers have extended this work by identifying regulatory fibroblasts that suppress joint inflammation during arthritis, driving the expansion of immune cell populations that actively contribute to the resolution of arthritis [93].

The phenotypic diversity of fibroblasts is driven by a combination of cell-intrinsic differentiation pathways, molecular signals from soluble factors, and cell–cell communication networks established by infiltrating immune cells within the tissue microenvironment [82,91,92]. Through multiomic spatial analysis of synovial tissue, we mapped the topography of the inflamed synovium in patients with inflammatory arthritis. This analysis identified distinct sublining tissue niches, each supported by spatially programmed subsets of synovial fibroblasts. Notably, we observed that perivascular fibroblasts in the sublining tissue activate specific ECM gene expression programs in response to cytokine signaling from the local microenvironment during inflammation. These changes create adapted tissue niches that either facilitate or restrict immune cell trafficking into the tissue.

Specifically, IFNγ-responsive fibroblasts create a pathogenic, immune-permissive niche that facilitates the recruitment and aggregation of lymphocytes within the tissue. In contrast, TGF-β-responsive, ECM-synthesizing fibroblasts produce a collagen-rich barrier (comprising types I and VI collagens) around blood vessels, restricting leukocyte migration. This barrier acts as an immune exclusion zone that promotes the resolution of tissue inflammation. These findings suggest that fibroblasts engage in ECM remodeling within the synovium in response to inflammation, attempting to “heal” the tissue by excluding immune cells and limiting fibrotic scar formation. Identifying and enhancing endogenous fibroblast-driven repair processes that restore and maintain homeostasis within the tissue could offer novel therapeutic strategies for reversing diseased tissue microenvironments and promoting a return to a healthy state.

## 6. Understanding the ECM in Liver Fibrosis—Paving the Way Toward Antifibrogenic Interventions

A new era of drug development is emerging in the field of steatotic liver disease, with the goal of halting and even reversing the progression of liver fibrosis. As fibrosis advances, it leads to cirrhosis, which is characterized by architectural changes in the liver and the formation of regenerative nodules of hepatocytes. These changes result in portal hypertension, impaired parenchymal function, and liver-related events that contribute to morbidity and mortality in end-stage liver disease. These events include upper gastrointestinal variceal bleeding; ascites; hepatic encephalopathy; and, ultimately, liver failure, collectively referred to as decompensated liver cirrhosis. Decompensation events are associated with poor survival, and apart from liver transplantation, no effective therapy currently exists. Therefore, there is a critical medical need to reverse the patient’s trajectory before cirrhosis develops and to prevent progression to decompensation once cirrhosis is established. While it has been shown that cirrhosis can be reversed with treatment of the underlying causes, the point of no return remains unclear. Halting progression to prevent decompensation can offer significant benefits for patient outcomes.

The use of ECM markers is currently being explored in clinical trials involving patients with metabolic dysfunction-associated steatohepatitis (MASH) who have progressed to liver cirrhosis. In these patient populations, clinical research has focused on specific pathways and corresponding mechanisms of action of pharmacological agents. One study examined weight-independent mechanisms of action, such as the direct antifibrotic effects of several fibroblast growth factor 21 (FGF-21) compounds, as well as newly Food and Drug Administration (FDA)-approved intervention Resmetirom. Another group of studies investigated weight-dependent drugs, including glucagon-like peptide-1 (GLP-1) agonists, GIP, and glucagon, which address the disease from a more metabolic perspective. Current data have demonstrated the effects of these drug groups on fibrosis, which can be evaluated using histological endpoints, such as fibrosis staging, as well as non-invasive tests, circulating markers, and imaging-based elastography. These methods assess liver injury and/or fibrosis, with the goal of quantifying the effects on ECM remodeling. Circulating markers include PRO-C3, enhanced liver fibrosis tests, AST, ALT, a fibrosis index based on four factors (FIB-4), APRI, and others.

During the hepatic disease session at the ECM2024 meeting, several key topics were discussed, leading to important conclusions. It was noted that the monitoring of the ECM using noninvasive tests may soon be ready for clinical application, with noninvasive ECM tests already being used in pivotal clinical trials [94]. Dr. Aleksander Krag and Dr. Diana J. Leeming presented data showing that ECM markers are prognostic for liver-related events, such as decompensation and mortality, in patients with advanced liver disease caused by metabolic dysfunction-associated steatohepatitis (MASH). Regarding drug-induced effects on liver fibrosis, Dr. Judith Ertle presented the positive outcomes of a GLP-1/glucagon dual agonist, survodutide, which acts mainly through a weight-dependent mechanism of action. In a Phase 2b study, 63% of patients with F1–F3 fibrosis showed improvements in liver fibrosis stage after 48 weeks of treatment with the highest dose of survodutide. In a second Phase 2 study, Dr. Ertle demonstrated that survodutide also showed effectiveness in improving cirrhosis, including reductions in liver stiffness, liver fat, PRO-C3, and enhanced liver fibrosis, indicating antifibrotic effects. Dr. Erik Tillmann presented findings on a weight loss-independent mechanism of action with FGF-21 drug efruxifermin. This drug was highly effective in reducing liver fibrosis, with 75% of patients with MASH and F2–F3 fibrosis showing improvements in liver fibrosis stage in the highest dose group at week 96 (Phase 2b). Dr. Tillmann also highlighted that ECM-related markers of fibrogenesis (PRO-C3), fibrolysis (CTX-III), and cardiovascular outcomes (PRO-C6) were modulated favorably in patients treated with FGF-21 compared to a placebo. Dr. Zvonko Milicevic presented the potential antifibrotic effects of triple agonist GLP-1/GIP/glucagon (retratrutide) in a subgroup of a diabetes trial focused on MAFLD. Significant reductions in PRO-C3 (up to −26.3%), AST, and ALT were observed, supporting its potential efficacy. Additionally, Dr. Detlef Schuppan discussed the application of ECM markers in understanding fibrosis reversal and the transition from fibrosis to liver cancer. He emphasized the importance of targeting fibrosis reversal in future drug trials and using biochemical markers of ECM degradation in combination with fibrogenesis markers to better understand the balance between these processes. The possibility of preventing HCC through fibrosis reversal was also raised. Finally, several posters presented at the meeting demonstrated that fibroblast activity markers such as PRO-C3, PRO-C6, and thrombospondin-2 are strongly correlated with poor prognosis in patients with chronic liver disease. Additionally, preclinical models—including precision-cut liver slice models, animal models (particularly the Novel Gubra Amylin NASH (diet-induced obesity) model), and liver-on-a-chip systems—were shown to be promising tools for future drug screening and mechanistic studies of liver fibrosis.

Dr. Michael Cooreman provided an overview of the role of peroxisome proliferator-activated receptor (PPAR) signaling in fibrosis across various organ systems, including the liver, kidneys, heart, and lungs. While most data on antifibrotic effects focus on PPARγ as a mediator of fibroblast activation, the other isoforms, PPARα and PPARβ/δ, are also implicated in fibrosis pathways through both indirect mechanisms (such as anti-inflammatory effects and reduction of oxidative stress) and direct actions. pan-PPAR agonist lanifibranor has demonstrated efficacy in modulating both metabolic–immune markers [95] and fibrosis [96] in patients with metabolic dysfunction-associated steatohepatitis (MASH). The balanced agonist activity on all three PPAR isoforms may explain the significant effect size observed in histological improvements in fibrosis [94].

In summary, the progression of fibrosis to cirrhosis and the subsequent complications, collectively referred to as decompensation events, are primary determinants of morbidity and mortality in patients with chronic liver disease. Thus, the ability to regress or prevent the progression of liver fibrosis offers the potential to significantly improve outcomes for patients with conditions like metabolic dysfunction-associated steatohepatitis (MASH), which poses a major and growing global health burden. Historically, the quantification of fibrosis and its progression or regression has relied on categorical histological staging of liver biopsy specimens. This method, while informative, is invasive and provides only semiquantitative measurements. However, recent advancements in the development of non-invasive markers of fibrosis—independent of sampling error and providing continuous variables—hold great promise for the clinical evaluation of novel pharmacological agents in registrational studies.

## 7. Liver Fibrosis—Cause or Consequence of Cancer

Hepatocellular carcinoma (HCC) is one of the world’s most common and fastest-growing cancers, with approximately one million new cases diagnosed annually. Prognosis remains poor, with a five-year survival rate below 20%. Notably, HCC rarely develops in a normal, nonfibrotic liver, highlighting the significant role of scarring and its associated microenvironmental changes in the cancer’s pathogenesis. Additionally, tumor-associated fibrosis, or desmoplasia, is a key feature not only of primary liver and bile duct cancers but also of pancreatic cancer, among others. As our understanding of the ECM in cancer biology deepens, the complex interaction between the stroma and tumor cells opens new opportunities to attenuate or prevent cancer development in the liver.

The association between HCC and viral hepatitis has been well recognized for decades. However, the link between HCC and fibrosis associated with metabolic dysfunction-associated steatohepatitis (MASH) has only gained recognition more recently. Interestingly, unlike cancers associated with viral hepatitis, at least one-third of patients with MASH-associated HCCs had not developed cirrhosis by the time of their HCC diagnosis [97]. This underscores the critical role of the ECM in creating a tumorigenic environment in chronic liver disease.

Hepatic stellate cells (HSCs) play a central role in fibrosis pathogenesis in the liver. These resident nonparenchymal liver pericytes undergo transdifferentiation, or activation, into myofibroblastic, fibrogenic cells during both acute and chronic liver injury. Notably, myofibroblasts may arise from various heterogeneous precursor cells, including portal fibroblasts and immune cells. Similar myofibroblasts contribute to fibrosis in other tissues, such as the lungs, kidneys, and heart. In the liver, HSC activation leads to a range of stromal changes, including not only the accumulation and modification of ECM components but also significant alterations in the immune microenvironment. Additionally, ECM-bound growth factors can be released by proteases associated with tissue injury, providing essential growth signals to both hepatocytes and HSCs. A key example is TGFβ1, a potent fibrogenic cytokine, which is typically bound to a protein complex that maintains its latency [98]. Upon release from the ECM, this complex activates latent TGFβ1, thereby promoting ECM production.

The transition of HSCs into myofibroblasts requires a substantial energy supply, which is provided through cellular autophagy, enabling the consumption of intracellular substrates. In vivo studies in mice have shown that inhibiting HSC autophagy holds therapeutic potential by depriving HSCs of the energy necessary for activation [99]. Additionally, autophagy in myofibroblasts may supply critical amino acids that support tumor cell growth [100].

Recent research has uncovered an autocrine signaling network among HSCs that emerges in advanced fibrosis [101]. This network, which drives “cold fibrosis” (i.e., fibrosis without significant inflammatory cells), may explain the progression of fibrosis in advanced MASH, even when hepatic fat and immune cells decrease in the later stages of the disease, including established cirrhosis. Importantly, the autocrine signaling network is driven by unique receptor–ligand pairs that become more dominant in advanced stages, offering novel targets for antifibrosis therapies.

HSC senescence has recently been explored as a potential driver of dysregulated immunity, increased inflammation, tissue damage, and procarcinogenic signaling. Markers of senescence are cell-type specific, and several cell surface proteins can identify senescent HSCs [102]. This offers new opportunities to specifically target and clear senescent HSCs. Indeed, experiments using chimeric antigen receptor (CAR-T) cells to deplete senescent HSCs have shown improved liver function, as measured by serum albumin levels, and reduced fibrosis in experimental models of liver injury [103]. While the clearance of senescent HSCs holds therapeutic promise, this approach must be carefully regulated, as complete depletion of HSCs can impair liver regeneration [104].

Finally, the stiffness and mechanical properties of the ECM not only activate HSCs but also promote HCC. The signals driving the impact of matrix stiffness on cellular responses are gradually being understood, with recent studies implicating discoidin domain receptor 1 (DDR1) as a specific ECM-associated cellular receptor. DDR1 can exclude immune cells from the carcinogenic environment, thereby preventing the immune-mediated clearance of tumor cells [105].

Overall, there is growing appreciation for the complex interplay of cells, the ECM, and soluble signals in generating a tumor-promoting stroma in cancer. Attention is now focused on understanding how this microenvironment promotes tumorigenesis, although the fibrogenic signals derived from tumor cells remain largely unknown. New tools and deeper insights into ECM biology hold the potential to enhance our understanding and accelerate the development of diagnostics and novel therapies to prevent or treat HCC, the most common cause of primary liver cancer.

## 8. Skin Diseases

The ECM is essential for the proper functioning of the skin, providing both its unique elasticity and tautness. It supports the firm adhesion of the epidermis (the outer epithelial layer) to the underlying dermis (the mesenchymal layer). The dermal ECM not only creates microniches and defines tissue geometry but also serves as an instructive element, directly guiding cellular behavior. Through these instructions, the ECM helps maintain tissue homeostasis, including inflammatory balance, and provides regenerative cues following injury. Real-world evidence of the ECM’s critical role in skin homeostasis can be observed in genetic disorders that affect the ECM. For instance, certain subtypes of epidermolysis bullosa and Ehlers–Danlos syndrome both result in chronic tissue fragility, impaired wound healing, and a tendency toward uncontrolled inflammation.

However, changes in the dermal ECM are also associated with many other conditions, including inflammatory skin diseases. In these cases, unlike genetic ECM diseases, it is challenging to distinguish whether ECM alterations are a cause or effect of the disease pathology. Nevertheless, alterations to the ECM can provide valuable insights into disease stage progression and help determine the patient’s eligibility for treatment response.

An essential difference between extracellular and intracellular proteomes is their turnover rates. Intracellular proteins are usually rapidly renewed, whereas the ECM is much longer-lived, with turnover rates generally spanning weeks or months to years or even exhibiting no replacement during a typical lifespan [106]. This leads to the accumulation of spontaneous modifications of the ECM, which can lead to altered degradation dynamics and impaired stability. It may also lead to the release of biologically active ECM-derived peptides that can promote tissue regeneration and inflammation [106].

From the perspective of a genetic disease, that is, recessive dystrophic epidermolysis bullosa caused by type VII collagen deficiency, Dr. Alexander Nyström discussed insights on ECM deficiency-induced injury and inflammation-driven fibrosis. He showed the importance of the interplay between proinflammatory immune cells and fibroblasts in the progression of fibrosis [107]. He discussed fibrosis, which was manifested by altered ECM organization and limited dependence on changes in protein abundances. Furthermore, he disclosed the role of the proinflammatory immunity in [107,108] re-educating dermal fibroblasts into profibrotic fibroblasts that expressed transmembrane serine peptidase dipeptidyl peptidase 4 (DPP4) [108].

One profibrotic action of DPP4 is to partially digest fibronectin, which alters the deposition of a fibronectin matrix that supports the deposition and assembly of interstitial collagen and fibrillin matrices [108].

Hidradenitis suppurativa (HS), also known as acne inversa, is a chronic inflammatory skin disease that manifests with progressive fibrosis of lesions. It has a prevalence of 1–4% in the general population. In HS, there is a skewing toward type I immunity [109]. Intertriginous body sites are predominantly affected, and despite the poorly understood pathogenesis and disease mechanisms, the key triggering factor is the occlusion of the hair follicle, caused by keratosis and hyperplasia of the follicular epithelium, leading to cyst development. Subsequently, the developed cyst ruptures, causing an immune response and inflammation that, depending on disease severity, may lead to abscess progression, sinus tract development, and skin fibrosis. Fibrosis develops in the dermis and epidermis, leading to irreversible scarring of the skin and excessive ECM destruction, which promotes further chronic inflammation.

Thus, ECM remodeling is highly active in HS pathogenesis [110]. Dr. Simon Francis Thomsen gave an overview of HS pathogenesis; current and emerging biologics for HS, which include TNF and IL17 monoclonal antibodies; and collagen-derived biomarkers in the serum as predictors of HS disease stage.

In another common inflammatory skin disease—atopic dermatitis—Dr. Dana Woerz described ECM remodeling and changes in ECM proteins at the skin level. She also alluded to connections with ECM remodeling, with a focus on asthma and changes in the lungs [111]. Dr. Hannah Paish presented on the development of improved full-thickness skin models for the testing of skin therapeutics, including antifibrotics.

Finally, in the session focused on dermatology, Dr. Alexander Eckersley discussed proteomic approaches to investigating age-related changes in the ECM through peptide location fingerprinting [112]. This method allows for the identification of peptides, whose exposure and susceptibility to proteolysis may change due to structural differences and/or modifications. Thus, the method can be used to extract information about the structural changes of ECM proteins that may occur because of damage, for example, through injury, inflammation, or aging.

Apart from the dermatology-focused session, numerous other dermatology-related presentations were featured at the conference. These included talks by Dr. Herve Pageon and Dr. Andrea Heinz on aging-related changes in skin, with a focus on spontaneous protein glycation and elastic fibers, respectively.

## 9. Systemic Sclerosis

Systemic sclerosis (SSc), or scleroderma, is a rare autoimmune disease characterized by a triad of pathogenic mechanisms, including (a) chronic vasculopathy; (b) autoantibody production, along with early inflammation because of activation of both innate and adaptive immunity; and (c) uncontrolled tissue repair leading to ECM accumulation and fibrosis. It is a heterogeneous disease with variable manifestations [113].

SSc is the rheumatic disease with the highest individual mortality rate and the most detrimental impact on quality of life. Although no disease-modifying therapies are available for overall SSc, several targeted therapies have been approved for the treatment of SSc-related manifestations.

Ongoing work has focused on its molecular heterogeneity (based on bulk RNA seq and single-cell RNA seq) and variable response to targeted therapies. Trials are incorporating this information as stratification or enrichment criteria [114]. In addition, there is increasing interest in cell-based therapies (autologous and allogenic) for autoimmune diseases. A chimeric antigen receptor (CAR) is a synthetic protein that, once introduced into an immune cell, can redirect the specificity of that immune cell against a specific target antigen [115]. Both T cells and natural killer cells are being engineered to target B cells (CD19) and plasma cells. Preliminary data have suggested efficacy in severe autoimmune diseases, including SSc, with acceptable toxicity. Ongoing Phase 1 trials will define long-term safety and durable response in this population. These trials will explore deep B-cell depletion in tissues and characterize the cellular and molecular events underlying fibrosis. They will also assess the arrangement of gene expression patterns mapped onto tissue sections to link structure and activity, allowing for the assessment of biological interactions at the cellular level. This will provide novel insights into the evolution of gene expression patterns over time with CAR-T, both in the affected skin and lymph nodes, as well as its impact on the ECM.

## 10. Cancer

During the progression of organ fibrosis and cancer, the ECM undergoes several significant alterations. These changes include modifications in its biochemical composition, increased cross-linking, linearized organization, and elevated degradation and turnover rates [116]. A dysregulated ECM is more than a structural scaffold; it plays a crucial role in modulating the various signaling pathways that drive disease progression.

For instance, the remodeled ECM alters biochemistry and biomechanics, in turn affecting cell adhesion, migration, and differentiation, which are critical processes in cancer and cancer metastasis. In addition, the structural (re)organization of the ECM [117] which is predominantly orchestrated by cancer-associated fibroblasts (CAFs), creates tumor-supportive microenvironments for tumor growth and spread. Furthermore, the dysregulated ECM contributes to an immunosuppressive microenvironment that enables cancer cells to evade immune surveillance, further aiding in their survival and proliferation.

### 10.1. Understanding ECM Changes Associated with Cancer

Dr. Raghu Kalluri (Professor, Department of Cancer Biology, the University of Texas MD Anderson Cancer Center, Houston, Texas) discussed the function of fibroblasts and collagen in organ fibrosis and cancer, focusing on a unique and rare homotrimeric form (α1/α1/α1) of type I collagen. Type I collagen is the most abundant protein in the body and is a heterotrimeric molecule consisting of α1/α1/α2 chains. The production of collagen 1 homotrimers in some cancers could be a maladaptive injury response induced by epithelial cells to survive. The production of type I collagen homotrimer by cancer cells in pancreatic ductal adenocarcinoma is driven by oncogenic Kras-induced suppression of the Col1a2 promoter by DNA methyltransferase (DNMT) 1. In addition to promoting cellular proliferation via the α3β1 integrin receptor, the type I homotrimer coats the cancer cells to suppress T-cell function and alter the local tumor microbiome, impacting the efficacy of immune surveillance and even immunotherapy [17]. The homotrimeric type I collagen variant represents an exciting new therapeutic target for immuno-oncology. Strategies for therapeutic intervention that are being explored for this include drugs that can block homotrimer formation or downregulate α3β1 integrin signaling, potentially blocking abnormal signaling, even in the presence of the collagen homotrimer.

Professor Janine Erler discussed the importance of ECM dysregulation during metastatic outgrowth and how it could be studied using decellularized organ scaffolds. Using mouse models of cancer and novel methods to isolate and decellularize specific organs, researchers can place the resulting ECM tissue scaffolds into special bioreactors to study cancer cell interactions with the tumor-associated ECM, focusing on the interactions regulating the colonization of distant metastatic sites. One previously developed methodology—in situ decellularization of tissues (ISDoT)—is well suited for high-resolution imaging and proteomic analysis [118]. This approach reveals premetastatic and metastatic niches that have an altered composition of ECM proteins, and ongoing studies are dissecting how these changes affect the colonization and outgrowth of cancer cells through intracellular signaling mechanisms [119]. This approach can be used to screen antimetastatic drugs that may prevent metastasis by normalizing premetastatic alterations and the metastatic niche.

### 10.2. Biomarkers in Solid Tumors

Professor Saurabh Gupta presented the pathological, prognostic, and predictive roles of circulating ECM biomarkers in patients with solid tumors and the lessons learned from randomized controlled clinical trials. ECM alterations influenced the response to treatment, including immune checkpoint inhibitors, and obtaining fresh biopsies was challenging. Circulating biomarkers were considered an easily accessible surrogate of therapy response. Data were presented on the application of noninvasive ECM and ECM-associated biomarkers to provide information on tumor biology, disease activity, and the identification of patients most likely to respond to checkpoint inhibition. Findings from both the Checkmate-040 hepatocellular carcinoma trial and the Checkmate-214 renal cell carcinoma trial were presented. Biomarkers of ECM formation, degradation, and TGFβ signaling were significantly increased in patients with HCC and advanced renal cell carcinoma compared with healthy individuals. High levels of ECM biomarkers were associated with poor overall survival and progression-free survival outcomes, with specific markers showing the predictive potential for an improved outcome with monoclonal antibodies nivolumab and ipilimumab, which block programmed death-1 (PD-1) and cytotoxic T-lymphocyte-associated antigen-4 (CTLA-4), compared with sunitinib, a multityrosine kinase inhibitor, in a renal cell carcinoma trial. Ultimately, these noninvasive ECM biomarkers could guide patient selection and stratification and were recommended for integration and systematic exploration in a pan-tumor setting, with an emphasis on evaluating how immunotherapies and anticancer drugs, in general, affect specific tumor ECM proteins and collagen signatures and, in turn, the immune cell compartment.

Dr. Nicholas Willumsen presented a comprehensive evaluation of the PRO-C3 serum biomarker assay and its potential role in prognostic enrichment and pharmacodynamic assessment, which could influence drug development. The PRO-C3 biomarker assay measures a specific peptide fragment released into the bloodstream during procollagen III processing and is associated with CAF activity and, consequently, tumor fibrosis [120]. Tumor fibrosis is prevalent in a subset of patients across various solid tumor types and is linked to aggressive tumor progression and poor overall survival. Thus, there is a pressing need to identify this “fibrotic” subgroup of cancer patients by developing and validating tools that assess tumor fibrosis-related risk parameters, which could serve as biomarkers in clinical cancer trials. In addition to PRO-C3, Dr. Willumsen presented data on tumor fibrosis, highlighting recent findings on collagen expression profiles specific to myofibroblasts (myCAFs), including type VIII, XI, and XII collagens [121]. When measured in serum, these collagen biomarkers demonstrated high diagnostic accuracy for cancer, prognostic value across multiple solid tumors, and significant interpatient variability, enabling stratification based on CAF activity and facilitating the monitoring of tumor fibrosis in patients. In summary, PRO-C3 and other myCAF collagen biomarkers offer a noninvasive stratification tool for patients entering clinical cancer trials. Utilizing fibrotic activity as a selection criterion may enhance response rates, improve the likelihood of drug development success, and spare patients from unnecessary treatments and toxicities.

### 10.3. Targeting ECM Changes in the Treatment of Cancer

Dr. Thomas R. Cox gave a presentation on the deconstruction of cancer ecosystems, focusing on lysyl oxidases (LOXes) as promising targets for stromal targeting in solid tumors. The LOXes are a family of five amine oxidases that are essential for the cross-linking and fibrillogenesis of fibrillar collagens. The progression of many solid tumors is accompanied by extensive fibrillar collagen deposition, which he showed could be exacerbated by standard-of-care therapies. As such, solid tumors typically exhibit a marked increase in lysyl oxidase (LOX) expression, which is associated with poor overall survival and relapse-free survival. Over the past two decades, several efforts have been made to develop inhibitors targeting various LOX family members, but these have yielded mixed results with often limited clinical success. Dr. Cox presented recently published work in collaboration with Sydney-based pharmaceutical company Syntara on the development and preclinical validation of SYN-5505, a novel, first-in-class mechanistic inhibitor of the entire LOX family [122]. The team demonstrated that SYN-5505 functioned as a bona fide antifibrotic when used in conjunction with chemotherapy in pancreatic ductal adenocarcinoma (PDAC). More importantly, they showed that chemotherapy triggered a robust desmoplastic response in tumors, leading to elevated tumor stiffness, decreased tumor perfusion, increased stromal activation, and heightened prosurvival signaling in tumor cells. Together, these factors blunted the efficacy of each successive round of chemotherapy. Crucially, SYN-5505 effectively blocked this chemotherapy-induced desmoplasia, significantly slowing tumor growth, reducing metastatic dissemination to the liver, and improving overall survival.

Dr. Marina Pajic presented a study on reprogramming of the profibrotic, immunosuppressive pancreatic cancer environment by repurposing antifungal itraconazole—an FDA-approved agent with potential anticancer properties—to enhance the overall antitumor response. The study employed single-cell RNA sequencing (scRNAseq) in an in vivo klebsiella pneumoniae carbapenemase pancreatic cancer model to investigate the stromal mechanisms by which itraconazole improves antitumor activity. The results indicated significant downregulation of the protumorigenic CD105+ CAF signature following itraconazole therapy, with a marked effect on myofibroblasts (myCAFs), which are responsible for the fibrotic desmoplasia characteristic of PDAC. These findings were supported by analyses showing decreased collagen deposition and altered ECM remodeling in itraconazole-treated tumors. Additionally, itraconazole treatment reduced metastatic spread to secondary sites. Enhanced immune responses were also observed, including an increase in proinflammatory macrophages and CD8+ T-cell infiltration. When combined with chemotherapy (gemcitabine/nab-paclitaxel) and immunotherapy, itraconazole significantly delayed disease progression. Notably, this combination therapy conferred significant survival benefits when paired with immunotherapy. These data provide a scientific rationale for further developing itraconazole in combination with immunotherapy and chemotherapy for pancreatic cancer, particularly in patients with protumorigenic and profibrotic CAF activity.

### 10.4. Future Directions

Alterations in the ECM during fibrosis and cancer are multifaceted and have profound implications for disease progression. By modulating key signaling pathways and fostering a protumoral, immunosuppressive microenvironment, a dysregulated ECM plays a central role in the persistence and progression of many solid tumors. Understanding these complex interactions is crucial for the development of targeted therapies that can disrupt these pathological processes and improve patient outcomes. Future research on the ECM in solid tumors should prioritize several key areas to further elucidate its role and therapeutic potential.

1. Further investigations into the specific signaling pathways and molecular mechanisms by which the ECM influences tumor behavior are needed. These should encompass ECM deposition and turnover, as well as the implications of changes in ECM architecture, biochemistry, and biomechanics.
2. A deeper understanding of how the ECM contributes to the immunosuppressive tumor microenvironment and influences the infiltration and function of various immune cells is needed to identify new strategies for enhancing immunotherapy efficacy.
3. Identification and validation of biomarkers that reflect ECM changes in tumors to enable early diagnosis, prognosis assessment, and monitoring of responses to both ECM targeting and conventional therapies are necessary.
4. The development and evaluation of novel therapeutic agents or the repurposing of antifibrotic drugs for cancer that specifically target ECM components or ECM-modulated signaling pathways should also be pursued. Potential approaches include the use of small molecules, antibodies, and gene therapies aimed at normalizing the ECM.
5. Further research is needed to investigate ECM variability not only across different tumor types but, more importantly, within distinct regions of the same tumor. Understanding how ECM composition correlates with cellular heterogeneity could enable the development of more precise, patient-specific therapeutic strategies.
6. Investigating how tumor ECM alterations contribute to drug resistance in cancer cells and tumors, as a whole, is crucial. Uncovering the underlying mechanisms will help identify strategies to overcome resistance and enhance the effectiveness of existing therapies.
7. Leveraging advanced technologies from bioengineering, imaging, and computational modeling will enable a more detailed study of the ECM. These approaches will provide new insights into its architectural, biophysical, and biochemical properties and their effects on cancer cells.
8. Advancing ECM-related discoveries from the laboratory to clinical trials will require innovative study designs that, for example, focus on high-tissue-formation endotypes. Such approaches will be crucial for evaluating the safety and efficacy of ECM-targeting therapies.

Collectively, advances in these areas have the potential to transform the treatment of cancer by exploiting the ECM in tumor progression and treatment efficacy.

## 11. Translational Mouse Models of Fibrotic Diseases: Where Do We Stand?

Despite significant advancements in antifibrotic therapeutic strategies, there remains a critical unmet need for more effective drugs targeting fibrotic diseases. One major challenge is the limited ability of preclinical models to fully replicate the features of corresponding clinical conditions. Selecting the appropriate preclinical model is a crucial step in drug development, as models that better predict the clinical efficacy of drug candidates are urgently needed. However, many current animal models lack a thorough characterization of their disease phenotypes, their relevance to human pathology, and their responses to clinically relevant drugs, raising concerns about their translatability and predictive value. Models with strong translatability can also aid in identifying novel biomarkers for fibrotic diseases. To enhance preclinical drug discovery, it is essential to establish robust and reproducible therapeutic outcomes in fibrosis models, necessitating the implementation of standardized replication protocols across laboratories.

Fibrotic disease stages are associated with distinct and shared biochemical, histological, and molecular mechanisms. Many models are limited by the development of early–intermediate fibrosis, in contrast to pivotal clinical trials in patients with more advanced stages of fibrotic disease. For example, “Western diet”-induced obese mouse models of MASH faithfully replicate metabolic, biochemical, and histological hallmarks of MASH and MASH-associated hepatocellular carcinoma but do not spontaneously develop cirrhosis, even when fed calorie-rich dietary regimens for up to 1.5 years [123]. Although “multiple-hit” models using nutrient-deficient diets, surgery, or toxin administration present fast-onset severe fibrosis, they have relatively low resemblance to the natural trajectory of the disease [124]. Many CKD models, including unilateral ureteral obstruction and renal ischemia–reperfusion injury (IRI), are useful in preclinical drug discovery, but they do not exhibit metabolic hallmarks consistent with diabetic kidney disease (DKD), which is the largest CKD patient group. To circumvent these limitations, a model of hypertension-accelerated advanced DKD has recently been developed using adeno-associated, virus-mediated renin overexpression in uninephrectomized diabetic db/db mice [125]. Most current models of heart failure with preserved ejection fraction (HFpEF) are limited in terms of their translatability because HFpEF is a multifactorial disease. Angiotensin-II infusion-induced hypertension models and leptin signaling-deficient obese/diabetic mice are widely used in preclinical heart failure research but do not fully recapitulate the clinical HFpEF phenotype [126,127]. In contrast, multiple-hit models combining angiotensin-II infusion in young or aged diet-induced obese mice may be an attractive option to better mimic the clinical condition and facilitate the development of HFpEF-targeted drugs [128].

Current IPF models only partially recapitulate the histological and molecular pathology of IPF. Although a single intratracheal bleomycin instillation in mice is the most frequently used IPF model, the model demonstrates variable lung fibrotic injury that gradually resolves to different degrees, depending on the strain of mice being used. This can be challenging when designing intervention studies and interpreting drug treatment outcomes [129]. Persistent lung fibrosis has been reported in mice following repetitive bleomycin installations and may enable long-term intervention studies [130]. Consistent with IPF primarily occurring in aged patients, aged mice have been reported to be more susceptible to bleomycin-induced lung fibrosis, showing impaired resolution [131]. Future studies should aim to clarify whether such model adaptations promote a consistent nonresolving phenotype and reproducible therapeutic outcomes. Adeno-associated virus (AAV)-driven pulmonary TGF-β1 overexpression and silica-induced fibrosis are valuable alternative murine IPF models [132].

Most intestinal fibrosis models develop fibrosis in the submucosa and muscularis propria layer, which is consistent with human pathology, but the overall degree of fibrosis is usually modest. The most frequently used models are not consistent with the inciting agents in IBD but, instead, use chemicals, such as dextran sodium sulfate or trinitrobenzene sulfonic acid, leading to epithelial damage and immune cell activation [133]. One recent model uses inoculation of the CD-associated bacterium adherent invasive E. coli, which induces colonic inflammation and fibrosis [134].

Another consideration lies in the choice of animal species for the disease model. Rats may exhibit a more pronounced fibrotic phenotype than mice. Although mini-pigs can also develop liver fibrosis, which reflects certain aspects of the human phenotype, long-term studies are needed to assess their applicability in preclinical drug development for MASH.

For any given fibrosis model, study cohorts display a heterogeneous disease stage, which can be a major source of variation in intervention studies. Although increasing group sizes may be useful for reducing variability, stratification to baseline disease severity allows for within-subject analysis of the treatment outcomes. In this regard, liver biopsy histology has proven to be highly instrumental in diet-induced obese MASH model studies [135] and noninvasive, unrestrained whole-body plethysmography allows for longitudinal monitoring of respiratory deficits and can complement terminal spirometry read-outs in IPF models [136]. Recent advances in preclinical imaging modalities, including micro-computed tomography (lung fibrosis burden), magnetic resonance imaging (application of collagen-targeted probes), micro-elastography (noninvasive assessment of fibrosis by measuring liver stiffness), PET (potential for fibroblast activation protein imaging; compound biodistribution), ultrasound imaging (noninvasive MASH and HFpEF assessment), and 3D light-sheet fluorescence microscopy (ex vivo mapping of fibrosis and drug biodistribution at single-cell resolution), enable visualization and quantification of whole-organ endpoints, helping gain knowledge about the heterogeneity of the disease model and treatment effects. Integrating imaging analysis with soluble biomarkers potentially offers an additional opportunity to improve the translatability of preclinical research to clinical applications. Recent advancements in mass spectrometry-based proteomics also allow for unbiased and hypothesis-free assessment of the plasma proteome, making them highly valuable in biomarker discovery. Beyond using these data to monitor the disease state of an organ over the course of an in vivo study, some biomarkers might even have the potential to be translated into the human setting and, thus, be used in clinical trials.

Considering that obesity and diabetes are major risk factors for the development and progression of MASH, CKD, and HFpEF, any model that combines the individual hallmarks of these fibrotic conditions could be highly useful in preclinical drug and biomarker discovery. Although an array of industry-standard obesity and diabetes models have been evaluated for their fibrotic comorbidities, they demonstrate low-grade or no liver, kidney, or myocardial fibrosis. Although this also applies to translational diet-induced obese MASH models, a Western diet MASH model with repeated low-dose carbon tetrachloride administration was recently reported to show indices of kidney injury resembling human CKD [137]. In addition, a rat model of severe pulmonary arterial hypertension—here, induced by vascular endothelial growth factor-2 blockade and chronic hypoxia—recapitulated concurrent liver fibrotic injury in chronic congestive right heart failure [31]. Collectively, the literature is sparse on models with multiple-organ fibrosis, and these models can also be challenging to develop and maintain. Further studies are needed to determine whether multiple-hit model strategies allow for the profiling of antifibrotic interventions in various organs in a single animal.

In conclusion, drug and biomarker discovery are highly innovative and competitive areas that bolster future hopes for diverse mono/combination therapies and diagnostic biomarkers for improving the outcomes of fibrotic diseases. To achieve this goal, cross-disciplinary approaches are required to establish, optimize, and validate fibrotic disease models for translatability and predictability.

### 11.1. In Vitro and Ex Vivo Human Models

In vitro and ex vivo human models are essential tools for studying ECM biology and testing the efficacy of antifibrotic or profibrinolytic compounds, as evidenced by numerous poster and oral contributions at the conference describing a variety of exciting models. Moreover, highly innovative 3D hydrogel matrices and organ-on-a-chip technologies were prominently featured in the commercial exhibition. The in vitro model platforms presented at the conference included 2D primary fibroblastic monolayer cultures in both diluted and macromolecularly crowded (pseudo 3D) culture media, 3D hydrogel cultures based on synthetic and tissue-specific decellularized ECMs, and the integration of these systems into organ-on-a-chip devices that enable mechanical stimulation and electric impedance measurements. Ex vivo skin, liver, and myocardial tissue slices, as well as full decellularized organs, were presented as effective tools for testing novel interventions aimed at modulating the quantity and quality of the ECM or fibroblast phenotype. A selection of conference contributions highlighting the variety and application of these innovations is presented below.

An awarded poster contribution by Birgit Cortes et al. (University of Bath, UK) and Boehringer Ingelheim was titled “Modeling a Micro-Niche with Tunable Hydrogels for the Characterization of Fibroblast Phenotypes in IPF”. The technique comprised stiffness characterization using nanoindentation of normal or IPF human lung fibroblasts encapsulated in synthetic vinyl sulfone-functionalized dextran (DexVS) 3D hydrogels. With respect to findings, the cross-linker concentration enabled the culturing of cells in hydrogels of different rigidities, impacting the fibroblast phenotype. Challenge with TGF-β1 or an IPF-related cocktail induced cells to adopt a disease-related phenotype compared with standard conditions, as characterized by an increase in *ACTA2* and *COLA1* expression. The study concluded that the culture of cells in the 3D hydrogel better recapitulates the IPF microenvironment and that the application of 3D hydrogels combined with the IPF-related cocktail will aid in the identification of novel drug targets specifically linked to disease-specific fibroblast phenotypes.

Further expanding the topic, in a Boehringer Ingelheim-sponsored oral presentation entitled “Modeling Fibroblast Heterogeneity In Vitro for Drug Discovery”, Vince Fiore demonstrated that the DexVS hydrogel platform, when blended with an electrospun fibrous scaffold, further increased the versatility of the 3D environment and its influence on fibroblast phenotypes. Notably, single-cell sequencing demonstrated that fibroblasts cultivated in a 3D environment and stimulated with TGF-β more accurately replicated the myofibroblast profile seen in IPF patients compared with fibroblasts cultivated in conventional 2D monolayer cultures.

A poster contribution by Karoline Mikkelsen (CelVivo and University of Southern Denmark) was titled “Utilizing a to Advance in Vitro Liver Fibrosis Modeling and Therapeutic Investigations”. The technique involved HSCs (LX2) being either monocultured or cocultured with hepatoblastoma cell line (HepG2) hepatocytes using a clinostat-based system to generate 3D spheroids. The 3D spheroids were subsequently treated with TGF-β to induce fibroblast activation. The activation of LX2 cells was assessed by quantifying hallmark fibrosis-related genes and biomarkers to confirm the induction of a fibrotic phenotype. With respect to findings, the formation of 3D spheroids was successfully achieved in both monocultured and cocultured conditions. Upon TGF-β stimulation, there was a notable increase in the expression of multiple collagen mRNA and elevated secretion of Nordic Bioscience fibrosis biomarkers of type I, III, and VI collagen, as determined by an enzyme-linked immunosorbent assay (ELISA). These effects were significantly reduced by the application of nintedanib, indicating its potential to mitigate fibrosis in this 3D model.

Further expanding on the topic, in a sponsored talk by Ectica Technologies, Benjamin Simona, in collaboration with Nordic Bioscience, presented the quantification of ECM biomarkers in the supernatants of primary cardiac fibroblasts growing in 3D poly(ethylene glycol) hydrogel precast plates used for high-content screening. ECM formation biomarkers increased upon TGF-β and PDGF-ββ stimulation and were dose-dependently inhibited by Omipalisib, an mTOR/PI3K inhibitor.

A poster contribution by Fabian Stavenuiter et al. (Charles River Laboratories) was titled “In vitro Modeling of Systemic Sclerosis for Drug Discovery Purposes”. The technique involved primary (myo-) fibroblasts being isolated from skin biopsies from patients with systemic sclerosis and healthy donors. A high-throughput screening campaign of more than 21,000 adenoviral shRNAs was conducted to determine changes in αSMA expression as a readout for the reversion of the myofibroblast phenotype. Fibroblasts were encapsulated in collagen hydrogels, and contractility was measured, along with impedance measurements, for target validation. With respect to the findings, several genes promoting the reversion of the myofibroblast phenotype in systemic sclerosis fibroblasts were identified. The collagen contractility cell-based assay was identified as a promising technique for validating these targets for drug discovery.

An oral contribution by Paola Occhetta et al. (BiomimX, Politecnico di Milano) was titled “Pathological Hallmarks of Human Cardiac Fibrosis in a Mechanically Active Organ-on-Chip to Predict the Efficacy of Drugs and Advanced Therapies”. The technique human atrial cardiac fibroblasts being embedded in fibrin hydrogels and loaded onto a mechanically active chip that enabled cyclic mechanical stimulation of the cultures alongside chemical stimulation. Cell proliferation and ECM deposition were characterized to monitor the progression of fibrosis in the presence of novel antifibrotic therapies. With respect to the findings, cyclic mechanical stimulation alone was sufficient to induce a fibrotic phenotype without the need for TGF-β stimulation, as evidenced by increased αSMA expression and ECM deposition. The value of this and other translational platforms lies in demonstrating the ineffectiveness of therapies that were effective in standard 2D monolayer cultures, helping to mitigate the risk of late failure.

An oral contribution by Hannah Paish (FibroFind and Newcastle University) was titled “Development of an Ex Vivo Full-Thickness Skin Model for Drug Testing and Disease Modeling”. The technique involved full-thickness skin biopsies (EVES) from healthy human skin tissue being prepared and maintained for up to 5 days in an ex vivo culture. This prolonged duration of culture allowed for the induction of inflammation or fibrosis through exogenous challenge with recombinant proteins. Moreover, the challenge of EVES with a disease-relevant psoriasis cocktail identified elevated secretion of disease-specific markers, including TSLP, IL-22, Granulocyte-macrophage colony-stimulating factor (GM-CSF), and IL-17, all of which could be attenuated by the IKK2 inhibitor. Critically, up to 300 EVES in a 96-well culture format or 150 EVES in a 24-well culture could be generated from each donor, providing a medium-throughput platform for the screening of numerous potential therapies in a single donor.

The implementation of new and emerging in vitro and ex vivo human models in preclinical research is critical for improving our understanding of ECM biology, helping to identify novel drug targets and allowing for the testing of novel therapies in models that closely resemble human disease. During the conference, 2D primary fibroblast cultures; innovative 3D hydrogel matrices; organ-on-a-chip technologies; ex vivo skin, liver, and myocardial tissue slices; and full decellularized organs were presented as effective tools for testing novel therapies. Twinned with these emerging models was the quantification of disease-relevant biomarkers to monitor the dynamics of the ECM and to better bridge the translational gap to clinical application.

### 11.2. Fibroblast Activation and Heterogeneity

A major focus at the congress was also the role of myofibroblasts as key cell types responsible for fibrotic progression and how these cells are activated [138]. Research into this biology and how activated myofibroblasts can be deactivated is likely to lead to effective antifibrotic treatments.

The distinction between hot and cold fibrosis was discussed at the congress. Hot fibrosis is characterized by high levels of immune cells (inflammatory macrophages). In healthy wound healing, a transient injury stimulates a brief inflammatory pulse that recruits a strong influx of macrophages, followed by an increase in myofibroblasts to the lesion. When the injury ends, both cell types disappear, resulting in a stable healing state with minimal ECM accumulation. In a hot fibrosis environment, the persistent presence of both myofibroblasts and macrophages in the tissue results in continuous activation due to the mutual secretion of growth factors and constant ECM production. Hot fibrosis occurs in pathological conditions with prolonged tissue injury from damaging agents or persistent pathogenic metabolic or inflammatory processes, such as chronic obesity or cancer.

In contrast, cold fibrosis is characterized by myofibroblasts without the presence of inflammatory macrophages. This state also leads to ECM accumulation due to the abundance of myofibroblasts. The intermediate-duration injury signals tend to lead to cold fibrosis. Inflammatory macrophages can both promote and abrogate the turnover of the ECM by secreting factors that enhance or inhibit ECM degradation (e.g., MMPs and TIMPs), which explains why fibrosis can occur both in the presence and absence of macrophages, depending on the context [139].

The obesity pandemic has led to an increase in cardiometabolic comorbidities, such as heart failure, stroke, diabetes, insulin resistance, and nonalcoholic fatty liver disease. The metabolic dysregulation induced by visceral adiposity leads to inflammation that drives fibroblast activation, resulting in increased levels of collagen secretion. General tissue fibrogenesis results in multiorgan impairment, which causes different disease indications characteristic of cardiometabolic disease [140].

Dr. Rachel Chambers reviewed her work on fibrometabolism in the context of IPF. Emerging evidence suggests that alterations in metabolism are not only a feature of the pathogenesis of fibrosis but may also play an influential role in it. In IPF, metabolic reprogramming modulates the fibrotic activities of lung fibroblasts. The inhibition of glycolysis, glutaminolysis, or arginine biosynthesis has been shown to reduce pulmonary fibrosis in animal models, suggesting that targeting metabolic reprogramming of fibrotic lung fibroblasts is a viable therapeutic avenue for this disease. Dr. Chambers has shown how TGFβ reprograms fibroblast metabolism to promote collagen biosynthesis via the mTORC1–4E-BP1 axis, which acts in cooperation with Smad3 to promote the production of cyclic AMP-dependent transcription factor ATF4. ATF4, in turn, orchestrates the subsequent transcriptional amplification of the glucose-derived serine–glycine biosynthetic pathway [141]. It is likely that the metabolic dysregulation seen in cardiometabolic disease also leads to fibrometabolism in organs other than the lungs.

Dr. Florian Rieder’s work on the intestine demonstrated the emergence of fibroblast heterogeneity in patients with fibrostenotic complications of CD. Fibroblast transcriptional profiles were examined using single-cell RNA sequencing of patient tissues with inflammatory or stricturing histologic subtypes. Several fibroblast populations were enriched in CD strictures compared with inflammatory or noninflammatory CD tissues. Intriguingly, by running CellChat analysis to computationally predict receptor–ligand interactions between neighboring cells, fibroblasts were found to be major signal senders within the tissue. Among these signal-sending populations, cadherin-11 (CDH11) was identified as a commonly upregulated gene that could potentiate cell–cell interactions between fibroblasts. Using genetic and pharmacologic inhibition in both in vitro and in vivo models of intestinal fibrosis, the role of CDH11 was validated in the promotion of fibrosis [31]. Furthermore, TLR5 ligand flagellin activated the inflammasome pathway in myofibroblasts to drive fibrosis, suggesting alternative fibroblast activation pathways with common outcomes in driving fibrosis. Finally, the ECM was shown to play an active role in intestinal fibrogenesis. Using a proteomic approach, the endogenous secreted glycoprotein milk-fat globule-epidermal growth factor 8 (MFGE8) was upregulated in CD strictures but, surprisingly, exerted an antifibrotic effect in vitro and in vivo through integrin signaling [142].

A further example of how ECM biophysical and biochemical properties influence fibroblast phenotypes was also presented. Fiore and colleagues described efforts to model fibroblast heterogeneity in vitro. Using a 3D hydrogel model that mimics the physico-chemical features of the interstitial matrix, including the fibrous mechanical cues and adhesive ECM ligands (including matricellular protein-mimicking Arginine–Glycine–Aspartic acid (RGD) and fibrous collagen-mimicking Glycine–Phenylalanine–Hydroxyproline–Glycine–Glutamic acid-Arginine (GFOGER) peptides), they showed that lung fibroblasts stimulated with canonical pro-fibrotic growth factor TGF-β resembled the transcriptional profile of IPF-specific myofibroblasts observed in patients. This was distinct from the transcriptional profile of fibroblasts cultured on typical 2D rigid substrates, which were significantly transcriptionally distinct from all in vivo populations. Importantly, this transcriptional profile included the upregulation of collagen triple-helix repeat-containing protein 1 (CTHRC1) and a signature of ECM proteolytic genes, including MMP11 and MMP13. In contrast, 2D cells upregulated a contractile signature in response to TGF-β. This supports the notion that fibroblast molecular phenotypes are qualitatively distinct between 2D and 3D culture settings, which motivated the further development of ECM-mimetic models that better recapitulate the tissue microenvironment. Furthering these efforts, the development of a coculture model with primary alveolar epithelial organoids in 3D models enabled direct cell–cell contact between alveolar stem cells and fibroblasts.

As another example of ex vivo models used to test therapeutic concepts in complex human cellular systems, Pliant presented a work that could differentiate the αvβ1/αvβ6 integrin inhibitors that block latent TGF-β activation from ALK5/TGFBRII inhibitors (ALK5i). Single-cell RNA sequencing analysis showed αvβ6 integrin upregulation specifically in aberrant basaloid cells and αvβ1 in CTHRC1+ myofibroblasts. Treatment of precision-cut lung slices with bexotegrast and subsequent single-nuclear RNA seq demonstrated that bexotegrast reduced TGF-β signaling and profibroblast gene expression (e.g., COL1A1, COL1A2, FN1, and POSTN), specifically in cell types with high target expression (i.e., aberrant basaloid cells and myofibroblasts). In comparison, ALK5i showed broad TGF-β signaling inhibition across all cell types, as expected for this molecule’s mechanism of action, suggesting a better safety/tolerability profile for bexotegrast. Overall, this represents an elegant approach to differentiating molecules in complex human models of disease, providing confidence in understanding the mechanism of action and the link between the target and the disease.

## 12. Proteomics

In this era of multiomics and big data, proteomics has proven invaluable for studying ECM biology in health, disease, and pharmacological treatment. This is particularly relevant to disease progression (e.g., fibrosis and cancer), where RNA expression may not reflect protein presence because of the longevity and permanence of tissue ECM components [112,143]. Similarly, for drug treatments in tissue, although next-generation sequencing (NGS) offers a sensitive way of capturing global cell responses, many cell types may synthesize matrix proteins but fail to deposit them into the extracellular space. As such, proteomic analysis offers an unbiased way to characterize the presence and abundance of ECM protein (i.e., the matrisome), which is crucial for biomarker discovery and understanding holistic changes in tissue proteostasis [144]. These standard approaches were put to good use by several presenting groups at this year’s ECM Pharmacology Congress. Using label-free liquid chromatography and mass spectrometry (LC-MS/MS), Woerz and colleagues from Berlin Institute of Health at Charité showed that psoriatic cytokines induce larger alterations in the skin fibroblast matrisome than those of atopic dermatitis. Colleagues from the University of Liverpool also employed labeled (Isobaric Tags for Relative and Absolute Quantitation (iTRAQ) and Stable Isotope Labeling by Amino Acids in Cell Culture (SILAC)) LC-MS/MS for more robust measurements of protein abundance to characterize the contribution of the ECM to the metastasis potential of uveal melanomas (Hattersley and colleagues and the new synthesis of ECM modifiers in fibrotic Dupuytren’s tissue (Canty-Laird)).

Over the past decade, the development of sample preparation methods, proteomic technologies, and bioinformatic databases (e.g., matrisomeDB for matrix protein classification and MatrixDB for ECM interactomes) has enabled a more robust analysis of ECM composition, protein abundance, degradation, and turnover [144]. Many of these developments were featured at the conference. Frattini et al. found that decellularization of mouse kidney led to improved ECM identification by LC-MS/MS but at the expense of higher intersample variability [145]. Despite this, decellularization remains a useful sample preparation method for ECM enrichment, as done by El-Merhie and colleagues from Justus Liebig University, who successfully identified the differences in chronic obstructive pulmonary disease (COPD) and healthy human lung tissue matrisomes. Alternatively, solubility fractionation [146] where samples are subjected to increasing stringencies of chemical and mechanical disruption, has also proven a valuable extraction method, as employed by Bamberg and colleagues from University of Colorado to identify changes in basement membrane and vascular proteins in the breast ECM of obese cohorts. New-generation mass spectrometers, which boast greater sensitivity (e.g., Thermo’s Orbitrap Fusion Lumos Tribrid™), have also been used by groups, such as Broadwin and colleagues from Brown University, who showed enhanced recovery of cardiac function in postinfarction mice that were intramyocardially injected with lab-grown 3D ECM particles. Novel machine learning approaches provided by software like Data-Independent Acquisition Neural Networks (DIA-NN) [147] have recently made data-independent acquisition (DIA) MS analysis more suitable for complex datasets, decreasing technical variability compared with data-dependent acquisition. These methods were used by Holstein and colleagues from German Cancer Research Center (DKFZ) to show HSC activation by TGF-β and Growth Arrest-Specific 6 (GAS6) (human growth inhibitor-specific 6)/AXL pathways in liver cirrhosis and cancer.

Matrix-assisted laser desorption/ionization tandem mass spectrometry (Matrix-Assisted Laser Desorption-Ionization (MALDI)-MS/MS) represents a significant advancement in proteomic analysis, allowing for the analysis of large biomolecules, making it useful for the identification and characterization of complex ECM components and their post-translational modifications. For instance, MALDI imaging mass spectrometry (MALDI-IMS) has been employed to spatially resolve protein distribution within tissue sections, providing insights into the ECM’s role in various pathological states, such as cancer metastasis and tissue fibrosis [148].

Affinity proteomics (in contrast to MS-based proteomics) is another emerging field in which technologies such as multiplexed proximity extension assays allow for highly specific quantification of, potentially, thousands of proteins [149]. Here, a marker is simultaneously detected by two antibodies linked to a DNA oligostrand, which is then amplified and detected by NGS. This type of assay (Olink^®^) was employed by Rohbeck and colleagues (GmbH) to identify inflammation biomarkers in response to GABA-A+ allosteric modulators for MASH therapy. New technologies for spatial proteomics were also showcased: Soetopo and colleagues from Boehringer Ingelheim used laser-capture microdissection to enrich separate compartments of the lung epithelium prior to MS to explore site-dependent, longitudinal changes in the lung matrisome during repair. Tompkins and colleagues from University of Manchester used imaging mass cytometry to compare 44 protein markers simultaneously across tissue sections, revealing the interplay between the matrix, B cells, and fibroblasts in COPD, asthma, and healthy lungs.

Looking to the future, several novel technologies have real potential to improve our understanding of ECM biology in health, disease, and treatment. Peptide location fingerprinting, as presented by Eckersley et al. (University of Manchester) for the unbiased identification of ECM protein modifications [112] could be used to screen for new biomarker ECM fragments (including bioactive matrikines) or as a method of systematically mapping identified fragments back to their sources. Degradomic approaches [150] such as those employed by Dinh and colleagues from University of Freiburg to show increased endogenous proteolysis of the ECM in muscle-invasive bladder cancer cohorts, could also be used to this effect. Dynamic SILAC, where light and heavy labels are pulsed (e.g., fed) into animal models at separate time intervals to enable the determination of protein turnover [151] could be used to investigate the runaway cycle of matrix synthesis and degradation in fibrotic diseases. Single-cell proteomics by mass spectrometry (SCoPE-MS) is also on the horizon, which would greatly enrich our understanding of cell–matrix interactions [152].

Hence, it is an exciting time to be at the crossroads of proteomics and matrix biology, the impacts of which on our understanding of disease and drug discovery are sure to be far-reaching.

## 13. ECM Autoimmunity

Autoimmunity is a process by which the immune system mistakenly targets the body’s own tissues. Autoimmune diseases encompass a range of conditions, from systemic diseases like systemic lupus erythematosus (SLE), systemic sclerosis (SSc), and RA, which affect multiple organs, to organ-specific diseases such as type 1 diabetes (T1D), IBD, and multiple sclerosis (MS). Currently, more than 80 different types of autoimmune diseases are known, comprising 5–10% of all diagnosed diseases in Europe [153,154,155]. They present a significant burden on healthcare systems, the economy, and patients’ quality of life through chronic management costs, reduced workforce participation, and the need for ongoing multidisciplinary care.

The ECM Congress 2024 highlighted the critical role of the ECM in both health and disease. Many autoimmune diseases, including RA and systemic sclerosis (SSc), are characterized by excessive tissue remodeling, which, together with chronic inflammation, can alter the composition of the ECM and expose hidden antigens, potentially triggering or exacerbating autoimmune responses. Fibroblasts, which are the primary cells responsible for producing and remodeling the ECM, contribute to pathogenesis by excessive deposition of matrix proteins, leading to tissue scarring and organ dysfunction, as seen in SSc. In addition, activated fibroblasts produce proinflammatory cytokines and interact with immune cells, exacerbating the chronic inflammation seen in conditions like RA and SLE; fibroblasts can also present autoantigens to immune cells, further driving autoimmunity. The Wednesday symposium, The Fibro-Inflammatory Axis: Fibroblasts and Tissue Destruction, included several interesting talks from industry and academia. Notably, Prof. Dana Orange (Rockefeller University) discussed the discovery of preinflammatory mesenchymal (PRIME) cells, which circulate in the bloodstream and appear to be the precursors to synovial fibroblasts—the cells that drive inflammation and joint damage in RA [156]. PRIME cells increase in number before a disease flare, suggesting that they could serve as early biomarkers of flare-ups. Prof. Adam Croft (University of Birmingham) highlighted the identification of distinct fibroblast subsets with either proinflammatory or anti-inflammatory properties (MMP3+/IL6+ and CD200+DKK3+, respectively) in the inflamed joint [157]. Recent findings by Dr. Anne-Christine Bay-Jensen’s (Nordic Bioscience) group showed that the interplay between growth factors (TGF-β and PDGF) and inflammatory cytokines (IL-1β and TNF) significantly enhances type III collagen production in fibroblast-like synoviocytes, underscoring the link between inflammation and fibrosis [158]. This was complemented by results from proteomic characterization of synovial tissue biopsies from longstanding patients with RA—presented by Prof. Allan Stensballe (Aalborg University) in the poster session—which identified unique molecular and cellular signatures underlying synovial pathotypes, suggesting that differences between lymphoid, myeloid, and fibroid pathotypes are continuous and linearly modellable. Altogether, these findings contribute to a deeper understanding of the complex interplay between inflammation, fibroblasts, and fibrosis in autoimmunity, underscoring the potential for developing targeted therapeutic interventions to modulate inflammatory and fibrotic processes.

The crucial role of ECM composition in tissue pathology and disease progression was emphasized in Prof. Raghu Kalluri’s presentation during the opening session. This presentation highlighted the dual roles of fibroblasts and collagen in fibrosis and disease. His findings on the altered structure of type I collagen in pancreatic disease revealed that a homotrimeric collagen structure composed solely of COL1α1 chains promotes cancer growth and proliferation by modifying the tumor microenvironment, presenting a novel potential therapeutic target [17]. It is possible that these structural changes in collagen could modify the immune environment in autoimmune disease; thus, a similar investigation would be valuable in the autoimmunity field to better understand disease progression and facilitate the discovery of new treatments. Dr. Kalluri’s recent research on pancreatic cancer also stressed the importance of leveraging evolving technologies to gain deeper insights into immune system changes and identify new therapeutic approaches for significant impact.

Novel approaches to detecting and monitoring autoantibody biomarkers in autoimmunity and other disease areas were presented by Dr. Molly Coseno from Sengenics at the industry-sponsored symposium titled Unraveling ECM Dynamics: Techniques, which was very well attended and quite interesting. The presentation covered challenges in biomarker discovery and introduced Sengenics’ revolutionary KREX^®^ protein microarray technology. This technology uses correctly folded, full-length, functional protein antigens, which are crucial for autoantibody-based assays, as more than 90% of antibodies recognize conformational epitopes that are only available if the protein is correctly folded. Dr. Coseno also highlighted the pivotal role of antibody biomarkers in chronic diseases. Real-life case studies were presented, showcasing the power of patient immunoprofiling in autoimmune diseases such as SLE and cancer.

Looking ahead, we anticipate a continued focus on identifying ECM-related biomarkers and integrating autoantibodies with orthogonal omics and traditional biomolecular screening approaches. The relevance of autoantibodies in disease remains a critical topic of discussion. These autoantibodies serve as early indicators of significant health changes, manifesting before clinical symptoms, making them unique and valuable candidate biomarkers. Embracing new technologies, such as those focused on autoantibody profiling, allows for novel insights into the “matrisome” and the identification of relevant biomarkers for decision making. Further discussion is required on the application of autoantibody subtyping to achieve a more detailed and individualized understanding of disease. The detection of anti-ECM autoantibodies may be regarded as the “other side of the coin” in ECM research, complementing the fact that autoantigens are targets for autoantibodies in autoimmunity (e.g., RA and SLE) and could help stratify patients into distinct subgroups.

For example, in SLE, autoantibody profiling identified distinct subtypes, which were subsequently validated across independent research groups, providing additional data to guide treatment selection strategies [159]. To educate the scientific community about the utility of autoantibodies in health and disease, it is essential to emphasize the best practices for designing clinical trials that incorporate autoantibody or biomarker screening. It was encouraging to see several speakers at the ECM Congress focus on this topic, marking an important step toward identifying new therapeutic targets and raising awareness about the significance of autoantibodies. Moving forward, we need to embrace new technologies and better understand the “matrisome” to identify relevant biomarkers for informed decision making.

## 14. ECM Biomarkers and Precision Medicine

### 14.1. Precision Medicine

The drug development industry is unified in its goal to achieve significant disease modification and clinical remission in areas with substantial unmet needs. Success is increasingly driven by identifying unique, unbiased, and proprietary targets, avoiding generic “me-too” targets that can be applied to broad populations. By integrating precision medicine strategies from the outset of drug development, it becomes possible to effectively identify novel targets. This approach focuses on identifying measurable, patient-specific characteristics, which enables the selection of treatments tailored to those most likely to benefit. Precision medicine leverages advanced technologies, including genomics, proteomics, and other multiomic approaches, to decipher the molecular mechanisms underlying each patient’s disease.

### 14.2. The Relevance of the ECM

**Figure 2:**
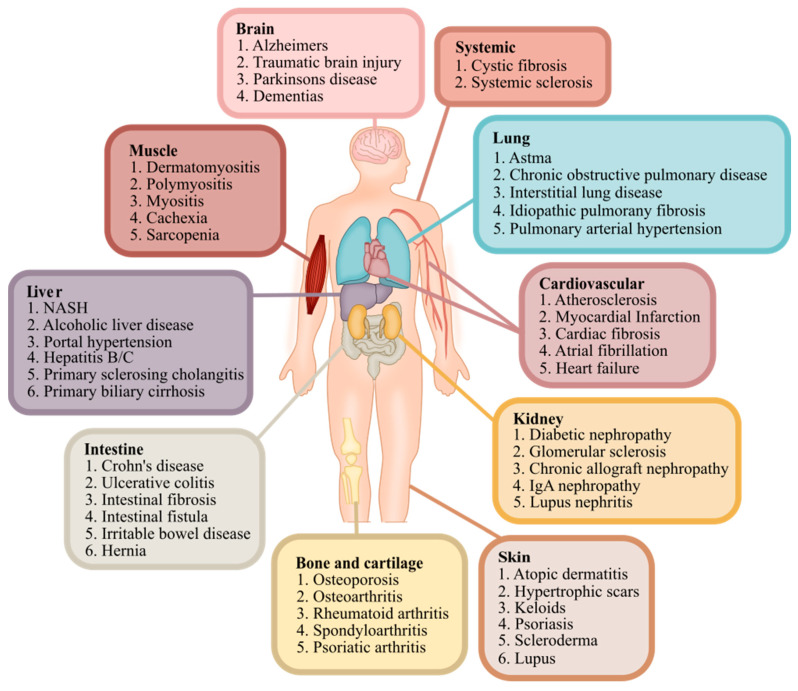
ECM remodeling is fundamental across chronic, usually fibrodegenerative, fibroinflammatory diseases. More than 50 different diseases have ECM remodeling as a common denominator. Modified from [160] with permission.

The ECM is a complex and dynamic network of proteins and other molecules that provides essential structural and biochemical support to surrounding cells. Its role is particularly significant in fibrosis, where fibroblast dysregulation affects ECM remodeling—a key factor in disease progression. The delicate balance between tissue repair, destruction, and inflammation within the ECM presents drug developers with valuable opportunities to utilize both prognostic and diagnostic biomarkers across a variety of diseases with significant unmet medical needs (Figure 2). A deep understanding of the ECM’s composition and its interactions with cells and tissues is crucial for the development of targeted, personalized therapeutic interventions. As outlined in Figure 2, the ECM is central to disease in more than 50 different pathologies (1). Furthermore, 35% of deaths in the Western world are associated with fibroproliferative diseases (2).

### 14.3. ECM Biomarkers

Since the discovery of beta-Crosslaps in the mid-1990s as a biomarker for bone resorption [161,162], they have become extensively used in both drug trials and clinical practice to monitor response and adherence. Beta-Crosslaps have since set the standard for the development of future ECM biomarkers.

For instance, in patients with metastatic melanoma, blood-based biomarkers of type III collagen turnover are linked to worse overall survival and progression-free survival following PD-1 inhibitor immunotherapy [121,163,164,165,166,167]. These ECM turnover measurements present opportunities for real-time patient stratification and diagnostics, enabling personalized treatment approaches. The integration of tumor-agnostic biomarkers with tumor-specific molecular indices may further enhance the accuracy of predicting immunotherapy responses in specific cancer types [168,169,170].

In RA, several ECM biomarkers resulting from the degradation of various collagen types (e.g., C1M, C2M, C3M, and C4M) and CRP (CRPM) have shown promise in influencing treatment decisions [171,172,173,174,175]. In one study, reductions in these markers within the first four weeks of tocilizumab (an anti-interleukin 6 receptor antibody) treatment helped distinguish between responders and nonresponders early on [60,70,174,176,177]. A similar trend has been observed with baricitinib, a Janus kinase inhibitor. Although ECM markers have not yet reliably predicted therapeutic responses before treatment, close monitoring of these markers and the resulting detectable reductions have been shown to precede clinical outcomes, suggesting their potential for early prediction of treatment success [60,70,174].

In IBD, a research partnership between AstraZeneca and the Crohn’s and Colitis Foundation has utilized real-world observational cohorts—known as IBD Plexus—to inform drug development at multiple stages. The integration of clinical, multiomic, and imaging data from over 2,800 adult and pediatric patients with IBD has provided several key insights:

Systemic IL-22 concentrations, associated with IL-23 pathway signaling in the gut, were identified as indicative of disease severity, influencing Phase 3 trial design and regulatory discussions.

The quality of genetically derived new targets improved, demonstrating downstream effects on circulating proteins.

In addition, ECM biomarker analysis has provided further insights into IBD pathogenesis and highlighted the emergence of ECM biomarkers as potential diagnostic candidates, such as CPa9-HNE, a calprotectin neoepitope, and C3-HNE, a neutrophil elastase-derived fragment of type III collagen [57]. Although the cohort sizes in these studies are modest, expanding to larger and better-phenotyped cohorts could deepen our understanding of ECM dysregulation and its relationship with underlying biology across IBD and other diseases [57,178].

ECM biomarkers also hold promise for the monitoring of disease progression across multiple conditions, with the use of panels to ensure tissue specificity likely being necessary. For example, a fibrosis algorithm incorporating age, diabetes status, PRO-C3 levels, and platelet count (ADAPT) has shown diagnostic performance that is either equivalent to or superior to the standard of care [179,180,181]. The potential of ECM biomarkers across multiple diseases could significantly increase the probability of success in biomarker development, potentially raising it from the current 10–20%, while also reducing development costs, which are currently estimated at USD 40–50 million, for bringing a new in vitro diagnostic test to market.

### 14.4. Outlook

The ECM Pharmacology Congress serves as a roadmap for collaboration, emphasizing the need to accelerate the development of novel treatments and diagnostics to address the unmet needs of patients. Through cross-industry innovation and engagement with academic and public–private consortia, several factors contributing to the success of precision medicine strategies have been identified. It is well established that ECM dysregulation plays a role in many disease pathologies, leading to pathological ECM turnover. Targeting these dysregulated processes with drugs has often shown clinical benefits, correlating with the normalization of ECM turnover. This area of research remains highly active, with ongoing debates about whether targeting fibroblast activity—along with upstream dysregulated molecular processes—could offer even greater therapeutic benefits.

## 15. Drug Discovery in IPF—How Can Biomarkers Support Key Decisions?

IPF is a chronic interstitial lung disease with an increasing incidence rate and a median survival of three years upon diagnosis. The current treatments for IPF—nintedanib and pirfenidone—slow the decline in disease progression but do not reverse it. These treatments also have well-established side effects, which can limit their use and acceptability among IPF patients. As such, there is a need to develop new therapies that offer a better side-effect profile and enhanced efficacy. These therapies need to be developed in the context of challenges including poor predictability of preclinical in vivo models, the requirement to conduct trials alongside current therapies, and the commercial challenge posed by these treatments being off-patent and relatively inexpensive.

Although there have been several notable failures in Phase 2 and 3 clinical studies, novel therapies are showing promise in late-stage trials. These include PDE4 inhibitor BI-1015550, LPA1 antagonist BMS-986278, and integrin antagonist PLN-74809. Furthermore, a range of novel therapies targeting the TGF-β and other pathways is in the early phases of drug discovery and development. The use of appropriate biomarkers will be crucial in advancing these potential therapies, alongside ex vivo translational studies with human tissue (e.g., precision-cut lung slices). These approaches can aid in drug development by facilitating target identification and validation, preclinical pharmacology studies, and translational research to assess early efficacy, ultimately leading to better clinical trial designs, such as improved patient selection.

In conclusion, exploring new targets in pathways other than TGFβ is essential to increase the potential for new and effective treatments for IPF. Biomarkers play a crucial role in guiding key decisions at all stages of the drug discovery and development process.

## 16. Imaging Techniques for ECM in Health and Disease

The ECM is composed of collagens, laminins, fibronectin, proteoglycans/glycosaminoglycans, elastin, and other glycoproteins. It serves not only as a structural scaffold that provides mechanical strength to tissues but is also highly bioactive, interacting with the body’s cells through various cell signaling pathways [182]. This 3D network is present in all tissues and organs, playing a critical role in maintaining tissue homeostasis and initiating wound responses in cases of injury or disease [182,183] such as tissue fibrosis, cancer, atopic dermatitis, HS, osteogenesis imperfecta, and CD. Given the ECM’s involvement in such a diverse array of diseases, researchers are increasingly recognizing the importance of studying the ECM in greater depth to improve our understanding of its role in disease progression and resolution. This summary briefly outlines the current techniques employed to image and probe the ECM, as well as the methods presented for imaging collagens during the ECM Congress 2024.

When investigating the mechanical or structural properties of the ECM (tensile and compressive strength of the ECM, structure, topography, etc.), scientists rely on atomic force microscopy (AFM), transmission electron microscopy (TEM), scanning electron microscopy (SEM), and second harmonic generation (SHG). AFM can visualize the structure of proteins (elastin, laminins, collagens, etc.), including the D-banding patterns of collagens, along with any disorganization or degradation of the proteins. In addition, AFM can probe the torsional deformation of tissues, the topography of a sample, adhesion forces, or changes in stiffness, making it a powerful tool for understanding the structure and mechanics of the ECM, especially in tumor microenvironments [184,185,186]. New technology developed by Optics11 Life used nanoindentations and micro-rheology to probe the mechanical properties of ECM-like hydrogels or ex vivo tissue in the fields of wound healing, fibrosis, and cancer. TEM imaging is useful for looking at the higher-order organization of ECM proteins within tissue samples and measuring the length or diameter of proteins within them. SEM is helpful for understanding the structure of ECM materials by providing information about the pore size or visualizing the matrix organization surrounding the cells [187]. SHG offers the ability to image fibrillar structures such as collagens, elastin, and fibronectin, which are all components of the ECM. SHG can be used for in vivo, in vitro, and ex vivo experiments [188,189] and is based on the contrast of the alignment of the filamentous proteins themselves, making it a useful tool for identifying regions of damage that result in lower contrast [190,191].

Histological staining of biopsies is the gold standard for staging the severity of fibrotic conditions (lung, liver, kidney, etc.) because they can easily visualize spatial–temporal information of the ECM during fibrosis. Determining the amount of collagen is a key indicator of fibrotic severity staging. Masson’s trichrome (MT), picrosirius red (PSR), van Gieson, and even hematoxylin and eosin staining are common stains for visualizing collagen in tissue sections [192,193,194]. Immunohistochemistry staining using collagen-specific antibodies (types I, II, III, IV, IX, etc.) is also routinely used but can have varying results because of batch-to-batch differences in the antibodies. 3Helix’s novel collagen hybridizing peptides (CHPs) can target molecularly damaged/denatured collagen because of disease progression in tissue samples and act as a prognostic marker [195] or they can be used as a pan-collagen stain to determine total collagen content, much like PSR or MT [195]. In addition, CHPs are species-agnostic because they specifically bind to denatured collagen chains that make up the collagen triple helix and can stain damaged collagen with high specificity, regardless of species or tissue type [195,196]. CHPs can even be used for live animal imaging to assess collagen turnover in animal models like in vitro diagnostic, multiple myeloma, and IPF [197,198]. For the first time ever in ophthalmology, researchers from Roche were able to visualize damaged collagen in the back of the eye in a subretinal fibrosis mouse model using the in vivo CHPs [199].

Moving away from the fundamental properties of the ECM and toward evaluation of a full tissue or organ, other imaging modalities and tools have become useful. Magnetic resonance imaging (MRI), PET, and computed tomography (CT) are used to investigate whole organs as opposed to the ECM. MRI is used for soft-tissue examination and can reveal evidence of tumors, blood vessel and/or soft tissue damage, and so forth, making it a valuable tool for diagnosing cancers and fibrotic conditions [199]. MRE has been extensively validated to assess tissue stiffness as a proxy for fibrotic conditions, especially in the liver [200]. MRI and CT have also been extensively used to assess organ volumes, which are clearly altered with various degrees of disease. An example of this is liver volumes, which are reduced with increasing grades of fibrosis, while, for instance, spleen volume increases as a result of increased portal pressure with increasing fibrosis grades. Therefore, the liver-to-spleen volume ratio is especially sensitive in monitoring changes in fibrotic grades [201].

PET provides the ability to quantify receptor expression and metabolic organ function in real time with the use of radiotracers, which help detect cellular changes in organs earlier than MRI or CT scans. Several PET tracers are currently in development for both fibrogenesis and fibrosis [202]. An example is the novel affibody-based PET tracer targeting platelet-derived growth factor beta (PDGFR-β) [203]. PDGFR-β is highly expressed on activated stellate cells in the liver and on fibroblasts in other tissues, such as lung and heart tissue. Because it is hypothesized that the downregulation of fibrogenesis, reflected in lower PDGFR-β expression, will inhibit disease progression and precede fibrosis regression, this biomarker may offer a much faster readout of antifibrogenic treatments than the assessment of fibrosis itself, which may take a very long time to resolve. The hypothesis is that the downregulation of fibrogenesis will translate into inhibition of disease progression and, eventually, regression of the fibrotic burden. PET also has the ability to assess fibrosis directly using peptides that bind directly to collagen [202]. Combining MRI and PET enables the simultaneous investigation of cellular states and downstream functions to provide a thorough assessment of organ status in both preclinical and clinical situations.

## 17. Translational Approaches to Directly Treat Liver Fibrosis and Liver Cancer

Primary liver cancer (75–85% hepatocellular carcinoma [HCC] and 10–15% cholangiocarcinoma [CCC]) is the sixth most commonly diagnosed cancer and the third leading cause of cancer-related deaths. In addition, 70–90% of all primary liver cancers develop in the context of advanced fibrosis and cirrhosis [204]. Combined with chronic inflammation, the altered ECM in advanced fibrosis promotes not only further fibrosis but also HCC/CCC development. An abnormal ECM conditions the immune environment in fibrosis and cancer, where mainly M2-type macrophages and fibroblasts are functionally modulated toward a profibrogenic and cancer-immunosuppressive phenotype [205,206,207]. Here, specific ECM signals, for example, which are mediated by integrins, as well as more general mechano-signaling from the ECM to the embedded cells, have been identified as key factors. In particular, enhanced ECM stiffness, combined with enhanced viscoelasticity, elicits cellular–intracellular signals of fibrosis and cancer progression [208,209,210,211]. Moreover, the ECM, with its hundreds of proteins, proteoglycans, and glycosaminoglycans, serves as a reservoir for fibrosis, angiogenesis, and invasion-modulating growth factors, such as TGF-β, VEGF, bFGF, PDGF-AB/BB, oncostatin-M (OSM), and hepatocyte growth factor, that are released upon remodeling of the stressed or damaged ECM [212]. Specific proteolytic fragments of the ECM—notably, of numerous collagens, like endotrophin (collagen type VI) and endostatin (collagen type XVIII)—are potent activators of (myo-)fibroblasts, leading to the suggestion of the ECM as an endocrine organ [3].

Finally, the ECM proteome of liver cancer and fibrotic livers displays unique quantitative compositions, with proteomic analysis of these tissues enabling, for example, the identification of certain collagen types as “the bad collagens of fibrosis or cancer” [3,212].

Due to its high perfusion, its central role in metabolism, and the phagocytic system, the liver is usually a prime target organ for systemic drugs and a prime target for novel nanoparticle therapies that can further redirect pharmacological agents to hepatocytes or nonparenchymal liver cells. High liver targeting has been demonstrated in vivo for small interfering RNA (siRNAs) and antisense oligonucleotides (ASOs), along with liver-specific nanocarriers and other nano-encapsulated pharmacological agents. There is an increasing number of hepatocyte-targeted therapies that can effectively treat systemic diseases, often because of a misfolded but dispensable or overexpressed hepatocyte gene. Examples include the use of ASOs to treat transthyretin amyloidosis, lipoprotein(a) overexpression as a strong cardiovascular risk factor, or apolipoprotein C overexpression in severe hyperlipidemia [213,214,215] and siRNAs to treat acute intermittent porphyria driven by overexpression of aminolaevulinic acid synthase, alpha1-antitrypsin misfolding causing lung and liver fibrosis and cancer, and lipoprotein(a) overexpression [216,217,218,219].

siRNA- and ASO-based therapies are even more attractive for addressing direct targets related to liver fibrosis, such as key molecules in the pathological ECM or overexpressed in fibrogenically activated (myo-)fibroblasts/hepatic stellate cells, which have historically been difficult to target using small molecules [220]. Moreover, when encapsulated in lipoplexes or biodegradable nanocarriers, they are prominently taken up by liver macrophages and (myo-)fibroblasts/stellate cells [221,222]. Conjugation to cell-specific ligands can further increase macrophage or (myo-)fibroblast specificity [206,223]. Notably, siRNA-mediated knockdown of one of the “bad collagens of fibrosis”, that is, collagen I [3] using Col1a1 siRNA-loaded lipoplexes or polymer nanoparticles, resulted in up to 95% Col1a1 knockdown in parenchymal and biliary liver fibrosis, reducing pathological collagen accumulation by 40–70%, even with late-onset treatment [216,217]. This therapeutic efficacy was accompanied by the downregulation of other fibrosis-related transcripts and the attenuation of fibrogenic immune cell activation, and it was comparable to a (myo-)fibroblast-specific inducible knockdown of Col1a1 [224]. Biodegradable nanocarriers could also redirect bisphosphonate alendronate, a drug that blocks osteolytic activity of osteoclasts (bone matrix-degrading macrophages) in patients with osteoporosis, from bone tissue to the liver, where it repolarized liver macrophages toward an antifibrotic phenotype [225].

Studies addressing other ECM-related targets are still needed but are coming into translational focus. An example is the c-Jun Proto-Oncogene (JUN) family of transcription factors, which has been implicated in driving fibrogenesis and cancer [226]. Here, siRNA- or ASO-mediated knockdown of c-JUN family members may attenuate active parenchymal and biliary liver fibrosis, as well as the growth of syngeneic HCC in mice. Further exploration and validation of direct antifibrotic (and cancer) therapies is on the way, including in combination with already established therapies.

Finally, with the advent of highly predictive serum markers of collagen and general ECM synthesis, degradation, and turnover, some of which are superb predictors of liver or other organ fibrosis, including cancer, the early prediction of therapeutic efficacy, for example, in Phase 1b clinical studies, will be possible, including personalized, noninvasive therapy monitoring [4,227].

## 18. Conclusions and Perspectives

The ECM is the central component of many chronic and acute diseases. We most likely need to change the ECM to develop truly efficacious treatments for organ diseases.

The ECM is both a scaffold and an instructive unit that modulates signaling pathways that drive disease progression. It is also a reservoir of bioactive fragments. It is essential to understand and quantify the cell–ECM interaction with respect to the progression and reversal of fibrosis [3].

ECM pharmacology is required to further this line of research, and it needs to be enabled by a range of tools and technologies. We summarize some of these important areas for discovery and development below.

1. Proteomic approaches to detecting disease-induced peptide fingerprints [228] (peptide location fingerprints identify species- and tissue-conserved structural remodeling of proteins resulting from aging and disease) are needed to identify new biomarker ECM fragments, including bioactive matrikines.
2. Going beyond collagen biomarkers, attention should be paid to the development of proteoglycans, elastin, and other ECM proteins (which play a role in fibrosis and inflammation) for the diagnosis of diseases and monitoring of disease progression and therapeutic efficacy. We need to enable clinical chemistry to separate tissue formation from tissue destruction, which can help in the discovery and modulation of individual paths for tissue formation and tissue destruction disorders.
3. Increasing the use of organs on chips as ex vivo disease models and moving away from cell culture on standard plastic dishes will increase in vivo likeness and improve target discovery and validation.
4. New microscopy approaches need to be developed to monitor ECM organization and its alteration in diseases. This will help to distinguish between the regenerative response of basement membrane fibrosis and the dangerous myofibroblast activation and production in the fibrillar dense collagen of the interstitial matrix that overgrows the parenchymal tissue.
5. Identifying new mechanisms/pathways could lead to drug repositioning through the establishment of a common denominator in organ fibrosis, allowing for an understanding of regenerating tissues.
6. Inhibiting hepatic stellate cells, i.e., myofibroblasts, may have both cancer and antifibrotic therapeutic potential. Moreover, drug resistance in solid tumor types may be conferred by fibroblast activity.
7. The development of drugs targeting specific disease stages (e.g., the autocrine signaling network driving “cold fibrosis”, i.e., lacking inflammatory cells) is driven by unique receptor–ligand pairs that are more dominant in advanced stages and represent novel therapeutic targets in liver fibrosis.
8. Recognizing the interplay between the ECM and immune cells is essential because both hot and cold fibrosis exist, as in solid tumors [229]. The ECM may play a role not only in immunity and inflammation but also in targeting and excluding specific cell types.

## Abbreviations

General and ECM-Related Molecules	
ADAPT	A PRO-C3-based score
AMP	Adenosine Monophosphate
COL	Collagen
ECM	Extracellular Matrix
ELF	Enhanced Liver Fibrosis Test
GAG	Glycosaminoglycan
LOX	Lysyl Oxidase
MMP	Matrix Metalloproteinase
PRO-C3	Marker of Type III Collagen Formation
PRO-C6	Marker of Type VI Collagen Formation
TGF-β	Transforming Growth Factor Beta
TIMPs	Tissue Inhibitors of Metalloproteinases
VEGF	Vascular Endothelial Growth Factor
Key Diseases	
CKD	Chronic Kidney Disease
COPD	Chronic Obstructive Pulmonary Disease
HFpEF	Heart Failure with Preserved Ejection Fraction
IBD	Inflammatory Bowel Disease
IPF	Idiopathic Pulmonary Fibrosis
NAFLD	Non-Alcoholic Fatty Liver Disease
NASH	Non-Alcoholic Steatohepatitis
MASH	Metabolic Dysfunction-Associated Steatohepatitis
OA	Osteoarthritis
RA	Rheumatoid Arthritis
SLE	Systemic Lupus Erythematosus
SSC	Systemic Sclerosis
UC	Ulcerative Colitis
Techniques and Technologies	
AFM	Atomic Force Microscopy
CT	Computed Tomography
DDA	Data-Dependent Acquisition
DIA	Data-Independent Acquisition
ELISA	Enzyme-Linked Immunosorbent Assay
MALDI	Matrix-Assisted Laser Desorption–Ionization
MRI	Magnetic Resonance Imaging
PET	Positron Emission Tomography
RNAseq	RNA Sequencing
SHG	Second Harmonic Generation
TEM	Transmission Electron Microscopy
Key Cells and Biomarkers	
CAFs	Cancer-Associated Fibroblasts
HSCs	Hepatic Stellate Cells
NETs	Neutrophil Extracellular Traps
